# Identification of novel hypermethylated or hypomethylated CpG sites and genes associated with anthracycline-induced cardiomyopathy

**DOI:** 10.1038/s41598-023-39357-2

**Published:** 2023-08-04

**Authors:** Purnima Singh, Liting Zhou, Disheet A. Shah, Romina B. Cejas, David K. Crossman, Mariam Jouni, Tarek Magdy, Xuexia Wang, Noha Sharafeldin, Lindsey Hageman, Donald E. McKenna, Steve Horvath, Saro H. Armenian, Frank M. Balis, Douglas S. Hawkins, Frank G. Keller, Melissa M. Hudson, Joseph P. Neglia, A. Kim Ritchey, Jill P. Ginsberg, Wendy Landier, Paul W. Burridge, Smita Bhatia

**Affiliations:** 1 Institute for Cancer Outcomes and Survivorship, University of Alabama at Birmingham, Birmingham, AL USA; 2 Department of Pediatrics, University of Alabama at Birmingham, Birmingham, AL USA; 3 Department of Pharmacology, Northwestern University, Chicago, IL USA; 4 Department of Genetics, University of Alabama at Birmingham, Birmingham, AL USA; 5 Department of Biostatistics, Florida International University, Miami, FL USA; 6 Department of Human Genetics, David Geffen School of Medicine, University of California, Los Angeles, CA USA; 7 Department of Population Sciences, City of Hope, Duarte, CA USA; 8 Children’s Hospital of Philadelphia, Philadelphia, PA USA; 9 Seattle Children’s Hospital, Seattle, WA USA; 10 Children’s Healthcare of Atlanta, Emory University, Atlanta, GA USA; 11 St. Jude Children’s Research Hospital, Memphis, TN USA; 12 University of Minnesota, Minneapolis, MN USA; 13 Children’s Hospital of Pittsburgh of UPMC, Pittsburgh, PA USA; 14 Department of Pathology and Translational Pathobiology and Feist-Weiller Cancer Center, Louisiana State University Health Sciences Center-Shreveport, Shreveport, LA USA

**Keywords:** Genetics, Cardiology, Oncology

## Abstract

Anthracycline-induced cardiomyopathy is a leading cause of late morbidity in childhood cancer survivors. Aberrant DNA methylation plays a role in de novo cardiovascular disease. Epigenetic processes could play a role in anthracycline-induced cardiomyopathy but remain unstudied. We sought to examine if genome-wide differential methylation at ‘CpG’ sites in peripheral blood DNA is associated with anthracycline-induced cardiomyopathy. This report used participants from a matched case–control study; 52 non-Hispanic White, anthracycline-exposed childhood cancer survivors with cardiomyopathy were matched 1:1 with 52 survivors with no cardiomyopathy. Paired ChAMP (Chip Analysis Methylation Pipeline) with integrated reference-based deconvolution of adult peripheral blood DNA methylation was used to analyze data from Illumina HumanMethylation EPIC BeadChip arrays. An epigenome-wide association study (EWAS) was performed, and the model was adjusted for GrimAge, sex, interaction terms of age at enrollment, chest radiation, age at diagnosis squared, and cardiovascular risk factors (CVRFs: diabetes, hypertension, dyslipidemia). Prioritized genes were functionally validated by gene knockout in human induced pluripotent stem cell cardiomyocytes (hiPSC-CMs) using CRISPR/Cas9 technology. DNA-methylation EPIC array analyses identified 32 differentially methylated probes (DMP: 15 hyper-methylated and 17 hypo-methylated probes) that overlap with 23 genes and 9 intergenic regions. Three hundred and fifty-four differential methylated regions (DMRs) were also identified. Several of these genes are associated with cardiac dysfunction. Knockout of genes *EXO6CB*, *FCHSD2, NIPAL2,* and *SYNPO2* in hiPSC-CMs increased sensitivity to doxorubicin. In addition, EWAS analysis identified hypo-methylation of probe ‘cg15939386’ in gene *RORA* to be significantly associated with anthracycline-induced cardiomyopathy. In this genome-wide DNA methylation profile study, we observed significant differences in DNA methylation at the CpG level between anthracycline-exposed childhood cancer survivors with and without cardiomyopathy, implicating differential DNA methylation of certain genes could play a role in pathogenesis of anthracycline-induced cardiomyopathy.

## Introduction

Anthracycline-induced cardiomyopathy is a leading cause of premature death in children with cancer^1^. The risk for cardiomyopathy increases with anthracycline dose^2,3^. Other risk factors include young age (< 5 years) at anthracycline exposure, female sex, chest radiation and presence of cardiovascular risk factors (CVRFs: diabetes, hypertension, dyslipidemia)^4,5^. The considerable inter-individual variability in the dose-dependent association between anthracycline exposure and cardiomyopathy risk suggest a need for a better understanding of the underlying molecular mechanisms. Several studies have examined the role of single nucleotide variants (SNVs) associated with anthracycline-induced cardiomyopathy^6–8^. There is emerging evidence to suggest that anthracycline-induced myocardial injury may be related to oxidative stress, mitochondrial damage, cardiomyocyte apoptosis, necrosis and autophagy^9^. However, there is paucity of information regarding the functional significance of most SNVs, precluding a clear understanding of the pathogenesis of anthracycline-induced cardiomyopathy, as well as hindering the development of potential preventive or therapeutic strategies.

Phenotypic traits are determined not only by genetic variants of coding sequences, but also by mechanisms that regulate how genes are expressed. Epigenetic modifications (DNA methylation, histone modifications and RNA-associated gene silencing)^10^ regulate gene expression, thereby contributing to the functional state of the genome. Indeed, aberrant DNA methylation plays a role in human disease, including de novo cardiovascular disease^11–16^. Epigenome-wide association studies (EWAS) have identified regions harboring variation in DNA methylation associated with disease phenotypes^17^. Recently, Robinson et al.^18^ showed that there is genome wide loss of DNA methylation and a gain in hydroxymethylation in endomyocardial biopsies from patients with early and late cardiotoxicity due to anthracyclines. While there is some evidence of an association between microRNAs and anthracycline-induced cardiomyopathy^19,20^, the contribution of epigenetic changes remains largely unexplored. In the present study, we examined the peripheral blood methylome at single CpG resolution in anthracycline-exposed childhood cancer survivors with and without cardiomyopathy to address this gap.

## Methods

### Study design and population

Study used a matched case–control design to understand the pathogenesis of cardiomyopathy in childhood cancer survivors. Participants were enrolled to a Children’s Oncology Group (COG) study ALTE03N1 (Key Adverse Events after Childhood Cancer). Thirty-one COG institutions (see supplement for full names) contributed participants to the study after obtaining approval from local institutional review boards. Written informed consent/assent was obtained from patients and/or parents/legal guardians. The University of Alabama at Birmingham Institutional Review Board (IRB-150115006) approved all experimental protocols and methods. All methods were performed in accordance with the ethical standards of University of Alabama at Birmingham Institutional Review Board and with the 1964 Helsinki Declaration. Cases consisted of childhood cancer survivors who developed cardiomyopathy. For each case, patients with no signs or symptoms of cardiomyopathy were randomly selected as controls from the same childhood cancer survivor cohort, matched on primary cancer diagnosis, year of diagnosis (± 5 years) and race/ethnicity. The selected controls also needed to have a longer duration of cardiomyopathy-free follow-up compared with time from cancer diagnosis to cardiomyopathy for the corresponding case. Participants provided peripheral blood in K_2_EDTA tubes for germline DNA. For this report, we restricted the participants to anthracycline-exposed non-Hispanic White patients to ensure homogeneous populations of sufficient size.

Cases fulfilled American Heart Association criteria for cardiac compromise by presenting with signs/symptoms (dyspnea, orthopnea, fatigue, edema, hepatomegaly and/or rales); or, in the absence of signs/symptoms, had echocardiographic features of left ventricular dysfunction [ejection fraction (EF) ≤ 40% and/or fractional shortening (SF) ≤ 28%]. Lifetime anthracycline exposure was calculated by multiplying the cumulative dose (mg/m^2^) of individual anthracyclines by a factor that reflects the drug’s cardiotoxic potential^21^ and then summing the results. Exposure to chest radiation was treated as a yes/no variable. Cardiovascular Risk factors (CVRFs) included the presence of any of the following: diabetes, hypertension, or dyslipidemia.

### Genome-wide DNA methylation analysis

DNA methylation status of 850,000 CpG (5'–C–phosphate–G–3') sites across the whole genome was analyzed using the Illumina HumanMethylation EPIC BeadChip arrays (Supplementary methods). The methylation score for each CpG was represented as a ‘β’ value according to the fluorescent intensity ratio [range: 0 (non-methylated) to 1 (completely methylated)]. Raw intensity data (IDAT files) were assessed for quality using BeadArray Controls Reporter (Illumina). Probe location and gene annotation used Illumina reference files (GRCh37/hg19).

Raw IDAT files were analyzed in R (v4.0.2, https://www.r-project.org/), using the Bioconductor packages minfi (v.1.34.0) and ChAMP (Chip Analysis Methylation Pipeline, v.2.18.3) for EPIC arrays^22,23^. Initial probe filtering steps used a threshold of p > 0.01 to remove 8511 probes with poor detection values. Next, we removed 2927 probes represented by < 3 beads, 2829 non-CpG probes, 95,316 probes that overlapped with SNVs, 24 probes that mapped to multiple locations and 16,593 probes that were on X or Y chromosomes^24^. Data were normalized using Noob^25^ for within-array normalization, correcting for background fluorescence and dye bias (Supplementary Fig. 1A). To account for DNA methylation differences due to cell-type composition in whole blood, we performed reference based deconvolution using FlowSorted.Blood.EPIC (v.1.6.1)^26^ (Supplementary Fig. 1B), and corrected the beta matrix in ChAMP. Following this, the singular value decomposition method (SVD) was used to detect significant components of variation, including valid covariates for statistical analysis. Champ.runCombat^27^ was used to eliminate technical and biological sources of variation from normalized and cell-type corrected methylation data (Supplementary Figs. 2 and 3). Following QC, 740,636 probes were analyzed in 104 samples (52 cases; 52 matched controls). The epigenetic age estimates, including Horvath’s^28^, Hannum’s^29^, DNAmAge, PhenoAge^30^ and GrimAge^31^ were obtained using an online calculator (https://dnamage.genetics.ucla.edu/new)^28^.

### Differential methylation analysis

Paired differentially-methylated probe (DMP) and differentially-methylated region (DMR) analyses were performed in ChAMP^23^, to identify differences in methylation that distinguished cardiomyopathy cases from matched controls. Default Bonferroni-corrected alpha threshold was set to < 0.05 in ChAMP.

### DAVID enrichment analysis

To further investigate the function of DMPs, we performed gene ontology (GO) and Kyoto Encyclopedia of Genes and Genomes (KEGG) pathway analysis using DAVID bioinformatics resource tool. Terms with kappa value > 0.5 were considered significantly enriched.

### Epigenome-wide association study (EWAS)

EWAS was performed to identify the association between DNA methylation at CpG loci and anthracycline-induced cardiomyopathy. Normalized, cell composition- and batch-effect-corrected ‘β’ values were used as the dependent variable. The following covariates were examined: age at primary cancer diagnosis, sex, age at study, anthracycline dose, chest radiation, CVRFs, DNAmGrimAge and PhenoAge. Collinearity statistics was used to filter covariates with a correlation coefficient > 0.7 (Supplementary Fig. 4). The following covariates were retained in the EWAS model: DNAmGrimAge, sex*age at study, chest radiation, age at primary cancer diagnosis squared and matching pair identifier. A series of linear regression models were built, one CpG locus at a time, with methylation β-values (0–1) as the dependent variable^32^ and cardiomyopathy case–control status as the key independent variable. Probes with a p-value < 10^–8^ were considered epigenome-wide significant associations. A Manhattan plot was generated for visualization of EWAS results (CMplot R package). We also used ‘CpGassoc’ R package to verify results obtained by the EWAS model^33^.

### Functional analyses

#### Criteria for prioritizing candidate genes for functional analyses

We used the following criteria to prioritize genes for functional analyses: (i) significant association of CpG probes with cardiomyopathy in paired ChAMP analysis (DMP, DMR) and EWAS, (ii) expression of genes harboring significant probes in adult human heart and hiPSC-CMs^34,35^, and (iii) mechanistic plausibility and published association with cardiomyopathy.

hiPSC line 19c3 generated from peripheral blood mononuclear cells from a healthy individual using the CytoTune-iPS 2.0 Sendai Reprogramming Kit (Invitrogen, A16518^36^) was used (Supplementary methods). To generate gene knockout gRNA expression vectors, one to two gRNAs targeting all splicing variants of the targeted genes were designed using an online CRISPR design tool (IDT) with high predicted on-target score and minimal predicted off-target effect. DNA oligos (IDT) encoding each gRNA with BbsI ligation overhangs were annealed and inserted into the BbsI restriction site of a pSpCas9(BB)-2A-Puro (PX459, Addgene 62988) plasmid (Supplementary Table 1A). The constructed gRNA expression plasmids were confirmed by Sanger sequencing (Eurofins) with the LKO1_5_primer (5′-GACTATCATATGCTTACCG-3′). Supplementary Table 1B includes sgRNA oligo sequences and sequencing primers. Details of the CRISPR/Cas9-mediated knockout of candidate genes are summarized in the Supplementary methods. We used qRT-PCR to assess candidate gene knockout (Supplementary methods and Supplementary Fig. 5). All PCR reactions were performed in triplicates (Supplementary Fig. 6) in a 384-well plate format using TaqMan Gene Expression Master Mix (Applied Biosystems, 4444557) in a QuantStudio 5 Real-Time PCR System (Applied Biosystems, A28140). Supplementary Table 2 summarizes TaqMan probes. Differentiation into cardiomyocytes was completed by using a hiPSC line expressing an exogenous TNNT2 promoter-driven Zeocin antibiotic selection resistance cassette for cardiomyocyte purification^37^ (Supplementary methods).

Day 30 hiPSC-CMs were treated for 72 h with doxorubicin (0.01–100 μM). Cell viability was assessed after 72 h using a resazurin assay. Fluorescence was measured using a VarioSkan Lux Multi-Mode Reader (Thermo Scientific). Data were analyzed using Excel and graphed using Prism 7.0 software (GraphPad) depicting standard dose–response guidelines. Data were presented as mean ± SEM. Comparisons were conducted via one way-ANOVA test, an unpaired two-tailed Student’s t-test, or F-test.

## Results

### Demographic and clinical characteristics

The case–control set included 104 non-Hispanic White, anthracycline-exposed childhood cancer survivors (52 cases; 52 matched controls). As shown in Table 1, the median age at primary cancer diagnosis for the cases and controls was 7.5 years and 10.7 years, respectively. Cases received a higher cumulative anthracycline dose (308 mg/m^2^ vs. 253 mg/m^2^, *P* = 0.04) and were more likely to have CVRFs (44.2% vs. 7.7%, *P* < 0.001). The median time between cancer diagnosis and cardiomyopathy was 6.3 years; controls were followed for a significantly longer period (median, 11.9 years, *P* < 0.001).

**Table 1:** Participant characteristics by case–control status.

Variables	Cases (N = 52)	Controls (N = 52)	*P*-value*
Age at primary cancer diagnosis (years)			
Mean ± SD	7.8 ± 5.4	9.3 ± 6.2	0.21
Median (IQR)	7.5 (3.3–11.5)	10.7 (3.3–14.1)	0.25
Age at enrollment (years)			
Mean ± SD	19.0 ± 8.6	21.1 ± 8.9	0.17
Median (IQR)	19 (13.8–22.3)	20.5 (16–24.5)	0.21
Sex (N, %)			
Female	28 (53.8)	22 (42.3)	0.24
Cumulative anthracycline exposure (mg/m^2^)			
Mean ± SD	308.5 ± 104.4	261.2 ± 135.9	**0.049**
Median (IQR)	308 (240–375)	252.5 (150–372.6)	**0.04**
Chest radiation (N, %)			
Yes	15 (28.9)	16 (30.8)	0.83
Cardiovascular risk factors (N, %)			
Yes	23 (44.2)	4 (7.7)	** < 0.001**
Primary diagnosis (N, %)			
Acute lymphoblastic leukemia	13 (25)	13 (25)	Matched
Acute myeloid leukemia	4 (7.7)	4 (7.7)	
Ewing sarcoma	10 (19.2)	10 (19.2)	
Hodgkin lymphoma	6 (11.5)	6 (11.5)	
Neuroblastoma	4 (7.7)	4 (7.7)	
Non-Hodgkin lymphoma	4 (7.7)	4 (7.7)	
Soft tissue sarcoma	2 (3.8)	2 (3.8)	
Osteosarcoma	5 (9.6)	5 (9.6)	
Wilms tumor	4 (7.7)	4 (7.7)	
Time from diagnosis to cardiac event for cases or time to enrollment for controls in years			
Mean ± SD	7.1 ± 6.1	12.3 ± 6.7	** < 0.001**
Median (IQR)	6.3 (1.3–11.3)	11.9 (6.8–16.6)	** < 0.001**

*SD* standard deviation, *IQR* interquartile range.

*Estimated using Chi-square and Mann–Whitney U test for categorical and continuous variables, respectively.

Bold values denote statistical significance at *P* < 0.05.

### Differentially-methylated probes

Of the 5,500 probes with an adjusted *P*-value of < 0.05, 32 probes showed an absolute difference in β-values ≥  ± 0.05 (Table 2 and Supplementary Excel, Table A); 22 of the 32 probes were associated with known genes. Of the 22 genes 17 (77%) are linked to heart diseases (Table 2). Ten genes (*SLC18A2/VMAT2, PDXK, SPTBN4, SYNPO2/CHAP, SLC9A2/NHE2, AHRR, GPR139, GNAO1, TRABD2A/TIKI1* and *MAP9*) harbored hypermethylated (in cases compared to controls) CpGs and twelve genes (*NIPAL2/SLC57A4, OR4D10, FARP1/PLEKHC2, ZFAND6, TEX9, SLMAP, HDAC9, POU6F2, RAPGEF6, FCHSD2, PLEKHN1* and *EXOC6B*) harbored hypomethylated (in cases compared to controls) CpG probes.

**Table 2:** Top-ranked paired differentially methylated probes (DMPs).

Probe ID	*P*-value	Δβ*	Chromosome position	Gene	Feature	UCSC (hg19) island name	Biological function and/or association with heart disease
CpG probes hypermethylated in cases compared to controls							
cg15417294	1.52E−04	0.09	chr10:119019957	*SLC18A2/VMAT2*	Body		Neurotransmitter transport. Murine knockout models and polymorphisms in humans are associated with cardiac arrhythmias and human cardiac sudden death syndromes^62^
cg19815989	1.25E−04	0.08	chr21:45161359	*PDXK*	Body		Pyridoxal kinase is in the critical region on chromosome 21 for congenital heart disease and has increased expression in the developing human Down syndrome heart^63^
cg13848826	2.45E−05	0.07	chr10:15476870	*–*	IGR		NA
cg18772399	6.24E−05	0.06	chr8:89478349	*–*	IGR		CpG and SNP rs1012116 associated with blood lipid levels^64,65^
cg27461310	2.71E−04	0.06	chr19:41037348	*SPTBN4*	5'UTR	chr19:41035100–41035440	Actin-binding and cardiac conduction. Primarily found at the cardiomyocyte intercalated disc. Loss results in increased fibrosis and decreased heart function^66^
cg18637832	2.99E−04	0.06	chr4:119890132	*SYNPO2*	Body		Actin binding muscle protein. Localized at the Z-disc of skeletal muscle cells and cardiomyocytes^49,50,67^
cg04297105	2.82E−05	0.06	chr2:103235258	*SLC9A2 /NHE2*	TSS1500	chr2:103235376–103236554	Na+ -H+ exchange (NHE) is a major mechanism by which the heart adapts to intracellular acidosis during ischemia and recovers from the acidosis after reperfusion^68^
cg06802630	1.30E−04	0.06	chr5:322735	*AHRR*	Body	chr5:320788–323010	DNA methylation of the aryl hydrocarbon receptor repressor is associated with subclinical atherosclerosis^69^
cg08053904	5.36E−05	0.06	chr16:20085897	*GPR139*	TSS1500	chr16:20084707–20085305	G-protein coupled receptors play a central physiological role in the regulation of cardiac function and are targeted for the treatment of hypertension and heart failure^70^
cg04369835	3.26E−04	0.06	chr5:322705	*AHRR*	Body	chr5:320788–323010	As above^69^
cg25752677	1.33E−04	0.05	chr16:56309206	*GNAO1*	Body		In animal models, increased expression of Gαo1, induced by attenuation of NRSF-mediated repression, plays a pivotal role in the progression of heart failure by evoking Ca^2+^ handling abnormality^71^
cg25325322	1.06E−05	0.05	chr2:85073715	*C2orf89 /TRABD2A/TIKI1*	Body		SNPs in the loci show significant associations with either Troponin I or T in GWAS^72^
cg24512097	1.16E−04	0.05	chr4:156298332	*MAP9*	TSS1500	chr4:156297601–156298094	Cell division, mitosis
cg23058405	1.95E−04	0.05	chr3:105072537	*–*	IGR	chr3:105072529–105072962	NA
cg00840433	1.34E−04	0.05	chr1:180182658	*–*	IGR		NA
CpG probes hyomethylated in cases compared to controls							
cg17241353	2.45E−04	−0.05	chr20:53868015	*–*	IGR		NA
cg21125179	1.21E−04	−0.05	chr2:72858044	*EXOC6B*	Body		Circular EXOC6B is a key biomarker for the diagnosis of HF^43^
cg07714001	4.37E−05	−0.05	chr14:42880359	*–*	IGR		NA
cg25742326	1.88E−04	−0.05	chr1:901449	*PLEKHN1*	TSS1500	chr1:894313–902654	Binds to cardiolipin (CL) in mitochondria. Protein downregulated in hearts of rodent model of T1DM^73^
cg05679760	7.45E−05	−0.05	chr11:72711174	*FCHSD2*	Body		Variants associated with cardiovascular events^74^
cg02683668	4.54E−06	−0.05	chr5:130844625	*RAPGEF6*	Body		Guanine-nucleotide releasing factor
cg11830489	3.47E−04	−0.05	chr7:39045208	*POU6F2*	5'UTR		Transcription regulation
cg02909936	1.05E−04	−0.05	chr7:18939140	*HDAC9*	Body		Epigenetic repression and transcriptional regulation. Variants associated with ischemic stroke and increased risk via promoting carotid atherosclerosis. HDAC inhibitors as antifibrotic drugs in cardiac and pulmonary fibrosis. Governs responsiveness of the Heart to stress signals and plays a role in Heart Development^75–77^
cg02828505	1.96E−05	−0.05	chr3:57875202	*SLMAP*	TSS1500		Regulator of cardiac function at the sarcoplasmic reticulum. Associated with human dilated cardiomyopathy. Emerging regulator of normal and abnormal cardiac excitation–contraction coupling^78–81^
cg05558609	1.93E−05	−0.05	chr15:56726855	*TEX9*	3'UTR		Differentially expressed and methylated in mice developing heart^82^
cg24017056	2.83E−04	−0.05	chr15:80366893	*ZFAND6*	5'UTR		Loci significantly associated with MetS-related components in T2D^83^ and role in insulin secretion^84^
cg15185986	1.16E−04	−0.05	chr13:98919378	*FARP1/PLEKHC2*	Body		Developmental protein, guanine-nucleotide releasing factor
cg14119788	2.43E−05	−0.05	chr11:59243571	*OR4D10*	TSS1500		Olfactory receptor
cg02737747	7.59E−05	−0.06	chr8:99264018	*NIPAL2/SLC57A4*	Body		Magnesium ion transport
cg15288329	5.60E−05	−0.06	chr6:170268691	*–*	IGR		NA
cg08993331	3.52E−04	−0.06	chr2:45310530	*–*	IGR		NA
cg23280506	1.24E−04	−0.08	chr17:14201938	*–*	IGR	chr17:14201726–14202052	NA

*IGR* intergenic region, TSS1500: 200–1500 bases upstream of the transcriptional start site, *3'UTR* 3′ untranslated region, *5'UTR* 5′ untranslated region.

*Ranked on methylation difference (Δβ) between cases *vs.* controls.

**Figure 1:**
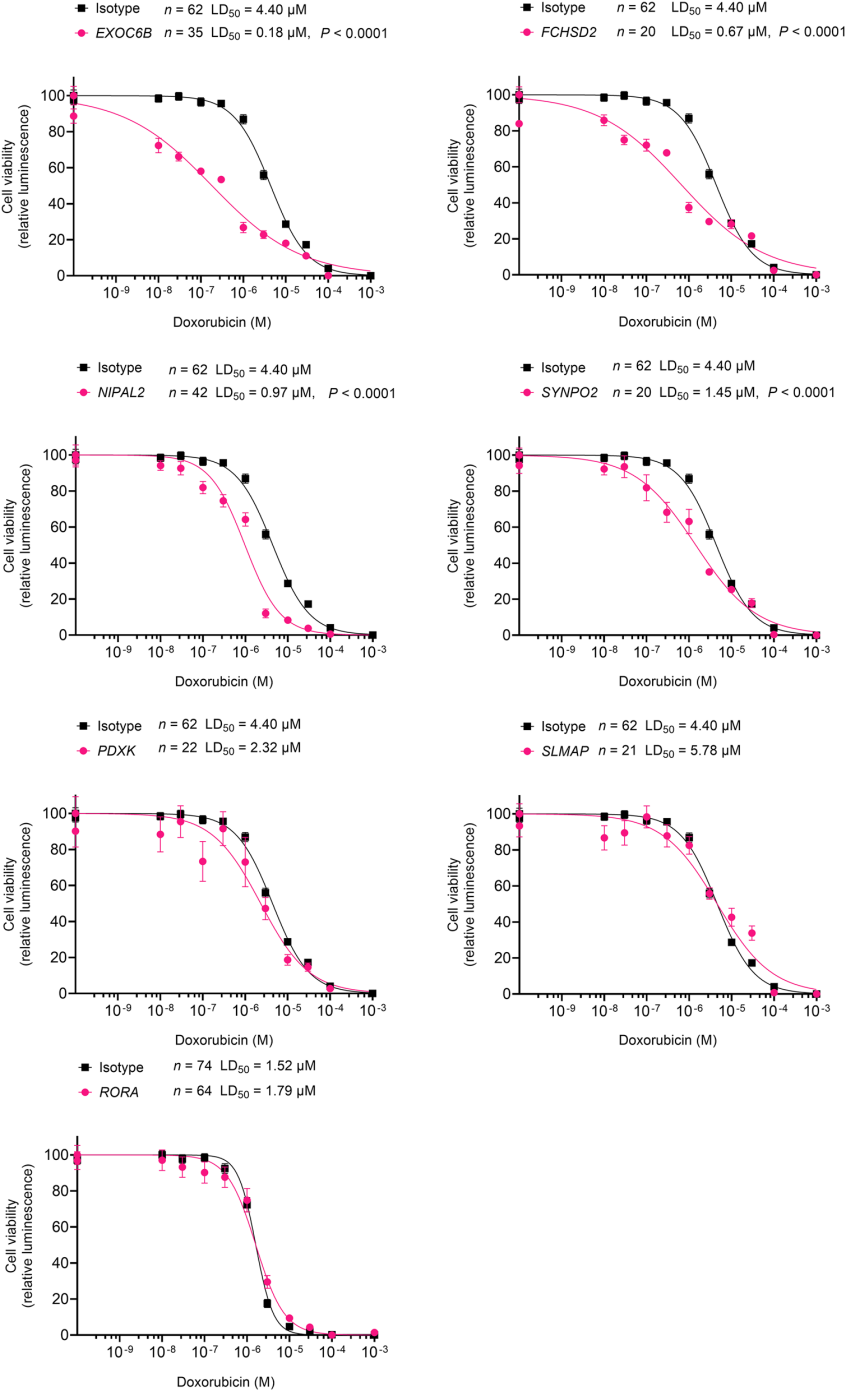
Assessment of in vitro anthracycline-induced cardiotoxicity in hiPSC-CMs. Effect of doxorubicin (72 h) on hiPSC-CM viability in control (isotype) and knockouts for *EXOC6B, FCHSD2, NIPAL2, SYNPO2, PDXK, SLMAP* and *RORA* are shown.

Of the 22 known genes that harbored DMPs, *EXOC6B*, *FCHSD2, NIPAL2, SYNPO2*, *PDXK*, and *SLMAP* are expressed in the adult heart tissue and hiPSC-CMs^38^. We examined whether the loss of function of these genes altered sensitivity to doxorubicin in an isogenic hiPSC line (ISO). Cell viability assays showed that the ISO-*EXOC6B* KO (LD_50_ = 0.18 μM), ISO-*FCHSD2* ko (LD_50_ = 0.67 μM), ISO-*NIPAL2* ko (LD_50_ = 0.97 μM), and ISO-*SYNPO2* ko (LD_50_ = 1.45 μM) hiPSC-CMs were 24-, 6.5-, 4.5-, and 3-fold more sensitive to doxorubicin as compared to ISO (LD_50_ = 4.40 μM, *P* < 0.0001), respectively (Fig. 1). *PDXK*, *SLMAP* and *RORA* KO- hiPSC-CMs did not show significant sensitivity to doxorubicin compared to the isogenic line.

The top hypermethylated (cg15417294) and hypomethylated (cg23280506) probes show a Δβ of 0.09 and −0.08, respectively (Table 2 and Supplementary Fig. 7). cg15417294 is associated with *SLC18A2/VMAT2* (Solute Carrier Family 18 Member A2/Vesicular Monoamine Transporter), whereas cg23280506 is located in the intergenic region and overlaps with a CpG island.

DAVID analysis showed a GADD_DISEASE_CLASS representation of “cardiovascular”, “chemodependency” and “metabolic” terms for genes that overlap with DMPs (Supplementary Excel, Table B).

### Differentially-methylated regions

Using the default *P*-value threshold of 0.05, we identified 354 paired DMRs (spanning 3 to 1396 base pairs) distributed over 1520 probes (Supplementary Excel, Table C); four probes were classified as DMPs in the above analysis. Overall, 33 DMRs were located on chromosome 1, 29 on chromosome 12, 27 on chromosome 6, and 23 each on chromosomes 2 and 5. The DMRs represented 323 annotated genes, of which 3 genes were included among the DMP genes above. The top two significant DMR-associated genes *HS3ST3B1* and *PNPO*/*SP2-AS1* are shown in Table 3 and Supplementary Fig. 8. Paired DMR_1 (chr17:14206572–14207968) was hypomethylated in cases and Paired DMR_2 (chr17:46018654–46019184) was hypermethylated in cases. Knockout of *PNPO* resulted in defective differentiation of hiPSCs to cardiomyocytes, suggesting a likely critical role of *PNPO* in cardiomyocyte differentiation and maturation.

**Table 3:** Differentially methylated regions (DMRs) associated with anthracycline-induced cardiomyopathy when comparing cases vs. controls with p-value area < 1 × 10^–5^ are shown.

Paired DMR	Chromosome position	Width (bp)	*P*-value area	Gene	Gene description	Biological function and/or association with heart disease
PairedDMR_1	chr17:14206572–14207968	1396	8.99 × 10^–5^	*HS3ST3B1*	Heparan sulfate-glucosamine 3-sulfotransferase 3B1	Differentially regulated during cardiomyogenesis^85^
PairedDMR_2	chr17:46018654–46019184	530	9.37 × 10^–5^	*PNPO/SP2-AS1*	Pyridoxamine 5'-phosphate oxidase	Catalyzes the terminal, rate-limiting step in the synthesis of vitamin B6. Vitamin B6 is a required co-factor for enzymes involved in homocysteine metabolism. High plasma levels of total homocysteine linked to increased risk of cardiovascular disease and stroke^86^. zPNPO deficiency causes cardiac disorders^87^

See Supplementary Excel, Table C for complete list.

### Epigenome wide association analysis

**Figure 2:**
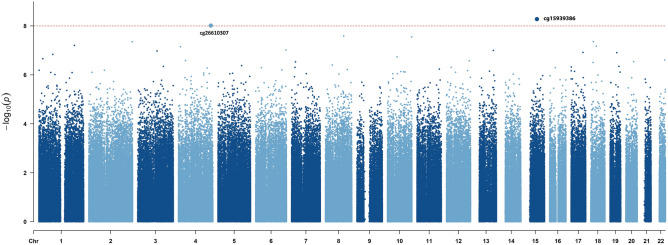
Manhattan plot of the epigenome-wide association study (EWAS) model. The *x* axis is the chromosome position, and the* y* axis is the significance on a –log_10_ scale. The red horizontal red line marks the threshold for the epigenome-wide significance (*P* < 10^–8^).

As shown in Fig. 2, two CpG probes exceeded the epigenome-wide significance threshold (*P* < 1 × 10^–8^); both were located in ‘open sea’. cg15939386 (*P* = 5.32 × 10^–9^) is located in the intron of *RORA* (Retinoic acid-related orphan receptor α) (Supplementary Fig. 9) and overlaps with distal enhancer-like signature EH38E1766996. The probe was hypomethylated in cases and had the smallest *P*-value 2.8 × 10^–8^ in the DMP analysis but showed a Δβ of −0.008 (cutoff for DMPs was ± 0.05). cg26610307 (*P* = 9.70 × 10^–9^) is in the X450k enhancer region, but does not overlap with any known genes.

## Discussion

We identified ten genes with hypermethylated DMPs and twelve genes with hypomethylated DMPs in childhood cancer survivors with anthracycline-induced cardiomyopathy when compared with survivors without. Seventy seven percent of these genes are linked to heart disease. Knockouts in hiPSC-CMs of four of these genes (*EXOC6B*, *FCHSD2***,** *NIPAL2*, and *SYNPO2*) demonstrated significantly increased sensitivity to doxorubicin. We identified 354 DMRs; *HS3ST3B1* and *PNPO*/*SP2-AS1* ranked as the top two DMR-associated genes. In the EWAS, we identified cg15939386 in the *RORA* gene to be significantly associated with anthracycline-induced cardiomyopathy.

Knockouts of DMP-harboring genes *EXOC6B*, *FCHSD2***,** *NIPAL2* and *SYNPO2* in hiPSC-CMs demonstrated significant sensitivity to doxorubicin, suggesting that these genes play a role in protecting cardiomyocytes from anthracycline-induced toxicity. Knockout of *EXOC6B* in hiPSC-CMs showed the highest (24-fold) sensitivity to doxorubicin compared to the control. *EXOC6B* encodes for the evolutionarily conserved exocyst, a multimeric protein complex necessary for exocytosis. It also encodes for a circular RNA cEXOC6B. Circular RNAs (circRNAs) are a class of long non-coding RNAs that play a role in cardiac hypertrophy, acute myocardial infarction, cardiac cell senescence, diabetic cardiomyopathy and heart failure^39–43^. Recently, a patent filed in 2018 (United States Patent Application 20200188356) showed that cEXOC6B was significantly dysregulated in left ventricular tissue in subjects with failing hearts compared to subjects with non-failing hearts. Although not well studied, there is evidence to suggest circRNAs can regulate gene expression by controlling methylation and that aberrant DNA methylation might regulate circRNA expression^44,45^. *FCHSD2* encodes a protein termed Carom that regulates cell growth, migration and adhesion^46^. *FCHSD2* also regulates F-actin polymerization, suggesting involvement in insulin exocytosis^47^. The locus has been associated with systemic lupus erythematosus^48^ which is frequently complicated by aggressive atherosclerosis. *NIPAL2/SLC57A4* is predicted to be involved in magnesium ion transport. *SYNPO2*/*Myopodin* is an actin- and α-actinin-binding member of the synaptopodin family^49^. It is localized in the sarcomeric Z-disc, but shuttles to the nucleus in cardiomyocytes in a stress- and differentiation- dependent fashion^50,51^. Pyle et al. proposed that complexes within the sarcomeric Z-disc drive downstream events in response to mechanical load of the heart, leading to cardiac hypertrophy^52^.

The top two DMR-associated genes were *HS3ST3B1* and *PNPO*. *HS3ST3B1* is downregulated in *rybp*^*−/−*^mice embryonic stem cells (ESC)^53^. Ring1 and Yy1 binding protein (Rybp) is a critical regulator of heart development and rybp null mice ESCs do not form normally functioning cardiomyocytes. In the rybp null cardiomyocytes, gene expression profiles revealed a downregulation of cardiac terminal markers. *PNPO* (pyridoxamine 5'-phosphate oxidase) is involved in the vitamin B6 metabolic pathway and is crucial to heart development during embryogenesis^54^. Zebrafish Pnpo morphants display a defective circulatory system^55,56^. Interestingly, cg19815989, a significantly hyper-methylated DMP in our analysis is on gene *PDXK* (pyridoxal kinase), also a key enzyme in vitamin B6 metabolism. Vitamin B6 is a required co-factor for enzymes involved in homocysteine metabolism. High plasma levels of homocysteine increase the risk of cardiovascular disease and stroke. Both *PDXK* (DMP) and *PNPO* (DMR) are hypermethylated in cases, possibly resulting in lower expression of these key genes with consequent aberrant vitamin B6 metabolism and increased risk of cardiovascular disease^57^. However, *PDXK* KO did not demonstrate sensitivity to doxorubicin in our study. Knockout of *PNPO* was incompatible with hiPSC differentiation to cardiomyocytes, indicating that this gene has a fundamental role in cardiac differentiation in human cells.

The CpG ‘cg15939386’ on *RORA* was significantly associated with cardiomyopathy in our EWAS model*. RORA*, also known as the nuclear melatonin receptor, plays an important role in the regulation of circadian rhythm, and protects against angiotensin II-induced cardiac hypertrophy and heart failure^58^. *RORA* is an endogenous protective receptor against diabetic cardiomyopathy by inhibiting oxidative stress and apoptosis in mouse models^59^. RORA protein is robustly expressed in non-failing human ventricular myocardium but is decreased in heart failure tissues, suggesting that RORA is a cardioprotective nuclear receptor^60^. lncRNA *RORA-AS1* (*RORA* Antisense RNA 1) has a regulatory role in the expression of *RORA.* The hypomethylated probe ‘cg15939386’ is present in an intron that overlaps both with *RORA* (‘−’ strand) and *RORA-AS1 *(‘+’ strand). In our study, hiPSC-CM knockout for *RORA,* however, was not differentially sensitive to doxorubicin compared to isogenic cells.

We need to consider the findings in the context of certain limitations. We did not consider other factors that affect methylation, such as socioeconomic status, health behaviors and environmental exposures. Our study focused only on non-Hispanic White survivors of childhood cancer. We performed methylation analysis in blood and not cardiac tissue. There is concern regarding the use of blood in epigenetic investigations in that it contains multiple cell types, each having a characteristic methylation profile. However, we performed a reference-based deconvolution to correct for proportions of different cell types in peripheral blood. DNA methylation patterns are often tissue-specific, and hence the concern that the peripheral blood may not reflect the methylation pattern in the heart. However, use of peripheral blood as a surrogate for cardiac tissue, has practical significance as ‘disease-affected tissues’ are not readily accessible for sampling. Meder et al.^61^ report cross-tissue conservation of epigenetic patterns occurring during heart failure. DNA methylation is thought to control transcriptional programs in a time-specific manner. We obtained samples from cases after they had developed cardiomyopathy. For future studies, it will be important to systematically evaluate DNA methylation markers in longitudinal cohorts of anthracycline-induced cardiomyopathy and heart failure.

## Conclusions

We performed the first epigenome wide analysis in peripheral blood derived DNA of childhood cancer survivors, with and without cardiomyopathy, following anthracyline exposure. We identified differentially methylated CpG sites and regions that are associated with genes that have previously been implicated in the pathogenesis of cardiovascular diseases and novel biologically relevant targets. Sensitivity to doxorubicin in hiPSC-CMs carrying the gene knock-outs of *EXOC6B, FCHSD2, NIPAL2, SYNPO2* need further investigation as potential mechanistic and/or therapeutic targets.

### Supplementary Information

Supplementary Information.

Supplementary Tables.

## Data Availability

The data discussed in this publication have been deposited in NCBI's Gene Expression Omnibus and are accessible through GEO Series accession number GSE224359 https://www.ncbi.nlm.nih.gov/geo/query/acc.cgi?acc=GSE224359.

## Abbreviations

CVRF: Cardiovascular risk factors
EWAS: Epigenome-wide association study
hiPSC-CM: Human induced pluripotent stem cell-derived cardiomyocyte
KO: Knockout
ISO: Isotype
LD_50_: Lethal dose 50

## Code availability

Scripts to perform reference-based deconvolution of whole-blood biospecimens using FlowSorted.Blood.EPIC package and correct the beta matrix in ChAMP have been deposited in a public GitHub repository and are available with the identifier https://github.com/lucylz/methylation.
