# Supplementary material for: Identification of novel hypermethylated or hypomethylated CpG sites and genes associated with anthracycline-induced cardiomyopathy
Source: Sci Rep. 2023 Aug 4;13:12683. doi: 10.1038/s41598-023-39357-2 (PMC10403495; doi:10.1038/s41598-023-39357-2)
Supplement: Supplementary file 1 — Supplementary Information. [file 41598_2023_39357_MOESM1_ESM.docx]

**Supplement**

**Identification of novel hypermethylated or hypomethylated CpG sites and genes associated with anthracycline-induced cardiomyopathy**

**Singh *et al.***

**Supplementary Methods**

**Supplementary Figures 1-4**

Supplementary Figure 1. Density plots show the distribution of the beta values

Supplementary Figure 2. Singular value decomposition (SVD) plot showing technical sources of variation in the data

Supplementary Figure 3. SVD plot showing biological sources of variation in the data

Supplementary Figure 4. Heatmap of Pearson correlation coefficient matrix

Supplementary Figure 5. Validation of CRISPR/Cas9–mediated KO for genes by Sanger sequencing

Supplementary Figure 6. Validation of KO by RT-PCR in hiPSC-CMs

**List of Participating Institutions**

**Supplementary Tables 1-2**

Supplementary Table 1. A. gRNAs with ligation overhangs used for generating CRISPR/Cas9 knockouts

B. List of primers used to validate CRISPR/Cas9-mediated genome editing using Sanger sequencing.

Supplementary Table 2. List of TaqMan probes used for RT-PCR to verify successful CRISPR/Cas9-mediated genome editing

**Supplementary Results**

**Supplementary Figures 7-9**

Supplementary Figure 7. Significantly differentially methylated probes (DMPs)

Supplementary Figure 8. Schematic representation of the top-ranked differentially methylated regions (A) *HS3ST3B1* (hypomethylated), and (B) *PNPO-SP2-AS1* (hypermethylated).

Supplementary Figure 9. Schematic representation of the top-ranked probe cg15939386 on *RORA-AS1*; *RORA* from the EWAS analysis

**Supplementary Excel**

Table A. List of Differentially Methylated probes (DMPs)

Table B. Functional interpretation of genes using DAVID bioinformatics resource

Table C. List of Differentially Methylated Regions (DMRs)

**Supplementary References**

**Supplementary Methods**:

**Genome-wide DNA methylation analysis**

Genomic DNA was isolated using Gentra Puregene Blood Kit (Qiagen Inc., Valencia, CA). DNA concentration was measured on Nanodrop ND-1000 Spectrophotometer (ThermoFisher Scientific Inc., Waltham, MA). DNA integrity was analyzed by Quant-iT™ PicoGreen™ dsDNA Assay Kit (ThermoFisher Scientific Inc., Waltham, MA). DNA methylation status of 850,000 CpG (5'—C—phosphate—G—3') sites across the whole genome was analyzed using the Illumina HumanMethylation EPIC BeadChip arrays. The methylation score for each CpG was represented as a ‘β’ value according to the fluorescent intensity ratio (range: 0 [non-methylated] to 1 [completely methylated]). GenomeStudio® software (v.2011.1; Illumina Inc., San Diego, CA) was used for extraction of DNA methylation signals from scanned arrays (methylation module v.1.9.0, Illumina Inc.). Raw intensity data were assessed for quality using BeadArray Controls Reporter (Illumina Inc.). Probe location and gene annotation used Illumina reference files (GRCh37/hg19).

Genomic DNA (1 μg) was bisulfite-converted using EZ DNA Methylation kit (Zymo Research Corp, Orange, CA, cat. no. D5004), and a cyclic denaturation step during the conversion reaction. Four microliters of bisulfite-converted DNA was used for hybridization on Infinium HumanMethylation 850 BeadChip. According to the Illumina Infinium HD Methylation protocol, the following steps were performed: whole-genome amplification, end-point fragmentation, precipitation and resuspension. The resuspended samples were hybridized onto HumanMethylation 850 BeadChips at 48°C for 18 hours. Then unhybridized and nonspecifically hybridized DNA were washed away, followed by a single nucleotide extension and repeated rounds of staining. Finally, the BeadChip was washed, coated and scanned. After scanning, the intensities of images were extracted using GenomeStudio Methylation module software (Illumina, Inc.).

***Human induced pluripotent stem cell derivation and culture***

Protocols and consent forms were approved by the institutional review board. Human induced pluripotent stem cell (hiPSC) line 19c3 was used throughout. This line was previously generated from peripheral blood mononuclear cells from a healthy control using the CytoTune-iPS 2.0 Sendai Reprogramming Kit (Invitrogen, A16518). Cells were routinely maintained in B8 medium on 1:800 diluted growth factor-reduced Matrigel (Corning, 356230). B8 was supplemented with 2 μM thiazovivin (LC Labs, T-9753), hereby referred to as B8T, for the first 24 hr after passage then fed daily with B8. Cells were passaged at a ratio of ~1:15-20 every 4days using 0.5 mM EDTA (Gibco*,* 15575020), achieving ~80% confluence. hiPSC cultures were maintained in 6 well plates.

***CRISPR/Cas9-mediated knockout of candidate genes***

hiPSCs were cultured in B8 medium to ~80% confluence. Cells were harvested using 0.5 mM EDTA for 6 min at room temperature and resuspended in B8T medium; 5×10^6^ cells were electroporated with 5 µg of each gRNA expression vector. Cells were maintained for 48h in B8T medium supplemented with 0.5 μg/mL puromycin (Gibco, A1113802). Puromycin resistant individual colonies were picked and expanded ~10 days after electroporation. Genomic DNA was extracted from the cell pellets using a Quick-DNA Miniprep Plus kit (Zymo, D4068). Clones with indels were identified by Sanger sequencing (Eurofins) with primers outside of the targeting region. Indels were detected using an online tool (https://benchling.com).

RNA was isolated using TRIzol reagent (Invitrogen, 15596026) and Direct-zol RNA microprep kit (Zymo, R2062) including on-column DNase digestion to remove genomic DNA. cDNA was produced from 2 µg of total RNA using a Maxima H Minus cDNA Synthesis Master Mix (Thermo Scientific, M1662). All PCR reactions were performed in triplicate in a 384-well plate format using TaqMan Gene Expression Master Mix (Applied Biosystems, 4444557) in a QuantStudio 5 Real-Time PCR System (Applied Biosystems, A28140). **Supplementary Table 2** summarizes TaqMan probes. Relative quantification of gene expression was calculated using 2^-ΔΔCt^ method, normalized to the reference 18S and untreated control samples.

***Cardiac differentiation***

Differentiation into cardiomyocytes was performed according to previously published protocol with modifications and using a hiPSC line expressing an exogenous TNNT2 promoter-driven Zeocin antibiotic selection resistance cassette for cardiomyocyte purification. Briefly, hiPSCs were split at a 1:15 ratio using 0.5 mM EDTA and grown in B8 medium for 4 days reaching ~75% confluence. At the start of differentiation (day 0), B8 medium was changed to R6C, consisting of RPMI 1640 (Corning, 10-040-CM), supplemented with 6 μM of glycogen synthase kinase-3 inhibitor CHIR99021 (LC Labs, C-6556). On day 1, medium was changed to RPMI, and on day 2 medium was changed to RBA-C59, consisting of RPMI supplemented with 2 mg/mL fatty acid-free bovine serum albumin (GenDEPOT, A0100), 200 μg/mL L-ascorbic acid 2-phosphate (Wako, 321-44823) and 0.5 µM Wnt-C59 (Biorbyt, orb181132). Medium was then changed on day 4 and then every other day with RBAI consisting of RPMI supplemented with 0.5 mg/mL fatty acid-free bovine serum albumin, 200 μg/mL L-ascorbic acid 2-phosphate and 1 µg/mL E. coli-derived recombinant human insulin (Gibco, A11382IJ). Contracting cells were noted from day 7, differentiated cardiomyocytes were treated with 25 μg/mL of Zeocin from day 10 to day 14. On day 20 of differentiation, cardiomyocytes were dissociated using DPBS for 20min at 37 °C followed by 1:200 Liberase TH (Roche, 5401151001) diluted in DPBS for 20 min at 37°C, centrifuged at 300 × g for 5 min, counted and plated onto Matrigel-coated 384 well plate for cell viability assay after doxorubicin treatment. Cardiac differentiation was done in 15 cm diameter cell culture dishes. 40,000 cardiac cells were plated per well of a 384-well plate in RBAI with 10% Cosmic calf serum (Cytiva, SH30087.03).

***Doxorubicin treatment and cell viability assay***

Doxorubicin hydrochloride (HY-15142, MedChem Express) was resuspended to 10mM in cell culture-grade water (Corning) and aliquots were stored at -20^°^C. Day30 hiPSC-CMs were treated for 72hr with doxorubicin (0.01-100 μM) diluted in RPMI 1640 medium (Corning) supplemented with 500 µg/mL recombinant bovine serum albumin (BSA) (GenDEPOT, A0100). Cell viability was measured after 72hr of doxorubicin (0.01-100 μM), using a Rezazurin assay. Resazurin sodium salt (Thermo, B2118703) was diluted to 5 mg/mL in sterile ultrapure water and 600 μL frozen aliquots were made. One aliquot was added to 14.4 mL of DPBS then further diluted 1:10 in RPMI. This was added to the cells 25 μL/well and incubated at 37 °C for 2.5 hr. Fluorescence was measured using a VarioSkan Lux Multi-Mode Reader (Thermo Scientific) using top read and an excitation wavelength of 560 nm and an emission wavelength of 590 nm. Data were presented as mean ± SEM. Comparisons were conducted via one way-ANOVA test, an unpaired two-tailed Student’s t-test, or F-test. The experiments were not randomized, and the investigators were not blinded to allocation during experiments and outcome assessment. Data were analyzed using Excel and graphed using Prism 7.0 software (GraphPad) depicting standard dose-response guidelines.

| **A**. Noob normalized data | **B**. Whole blood cell type corrected data |
| --- | --- |
| 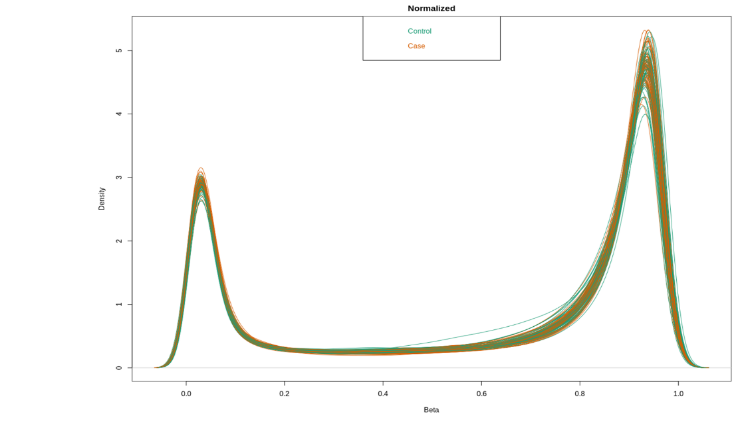 | 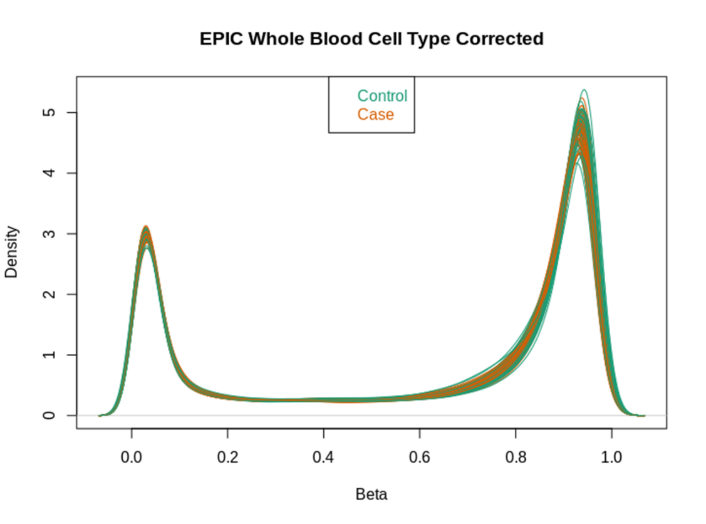 |

**Supplementary Figure 1**. The density plots show the distribution of the beta values for each sample type after normalization. The methylation levels of the 52 cases (green) and the 52 controls (red) are shown. **(A)** **Noob normalized data** **(B)** **Whole blood cell type corrected data**

| **A.** SVD prior to correction |
| --- |
| **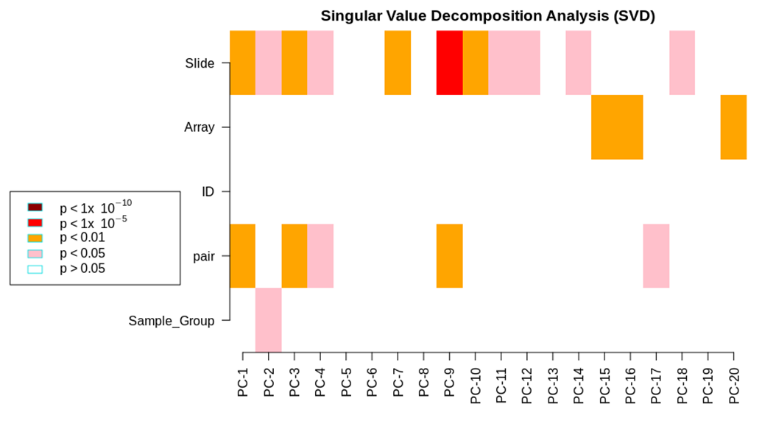** |
| **B.** SVD after correction |
| 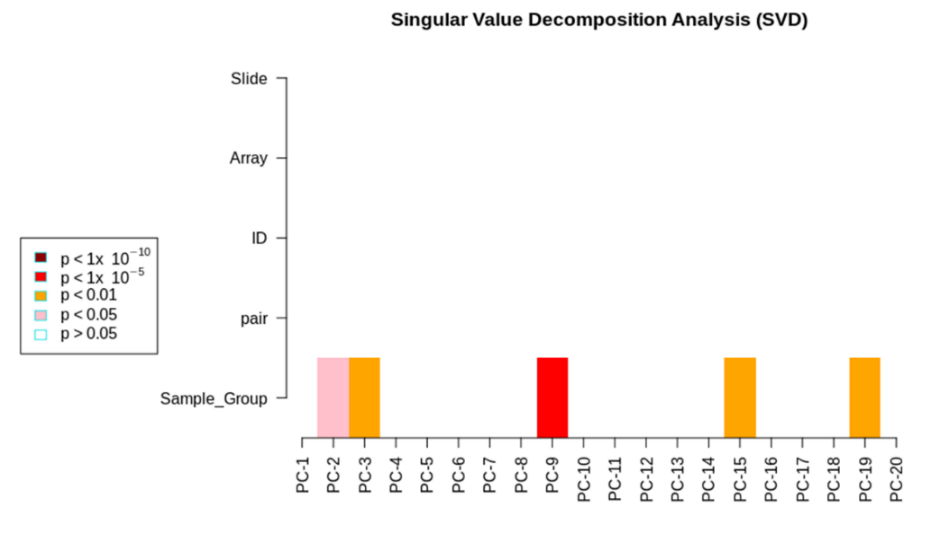 |

**Supplementary Figure 2.** Singular value decomposition (SVD) plot showing technical sources of variation in the data. Correction was made for array and slide with ComBat normalization. Color depth represents different significance levels (dark red: p < 10^-10^, red: p < 10^-15^, orange: p < 0.01, pink: p < 0.05, white: p>0.05). **(A)** **SVD prior to correction** **(B) SVD after correction**

| 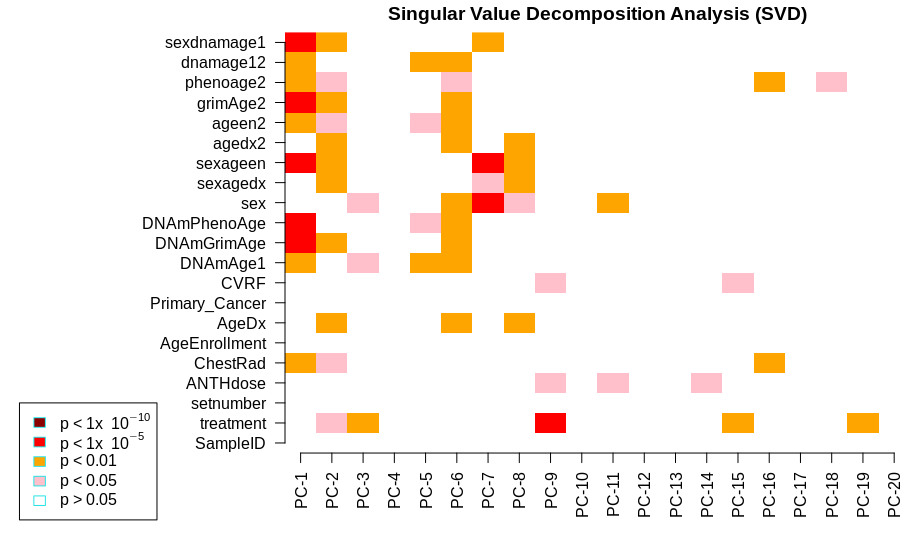 |
| --- |

**Supplementary Figure 3**. **Singular value decomposition (SVD) plot showing biological sources of variation in the data**. The association of each principle component with Biological/Epidemiological factors is indicated using *P* values. Biological/Epidemiological factors included age at the time of study enrollment (AgeEnrollment), interaction between sex and methylation age (sexdnamage1), methylation age squared (dnamage12), pheno age squared (phenoage2), grimAge squared (grimAge2), age at the time of study enrollment squared (ageen2), age at the time of primary cancer squared ( agedx2), interaction term between sex and age at the time of study enrollment (sexageen), interaction term between sex and age of the primary cancer (sexagedx), gender (sex), pheno age (DNAmPhenoAge), Grim Age (DNAGrimAge), methylation age (DNAmAge1), CVRF, Primary Cancer (Primary_Cancer), Age at the time of primary cancer (Agedx), Chest Radiation (ChestRad), cumulative Anthracycline dose (ANTHdose), matched case-control set number (setnumber), cardiomyopathy (treatment), Patient ID (SampleID). The darker blocks show stronger association between deconvoluted components (PC-1 to PC-20) and covariates.

| **A.** Correlation matrix heatmap with all variables | **B.** With selected variables |
| --- | --- |
| **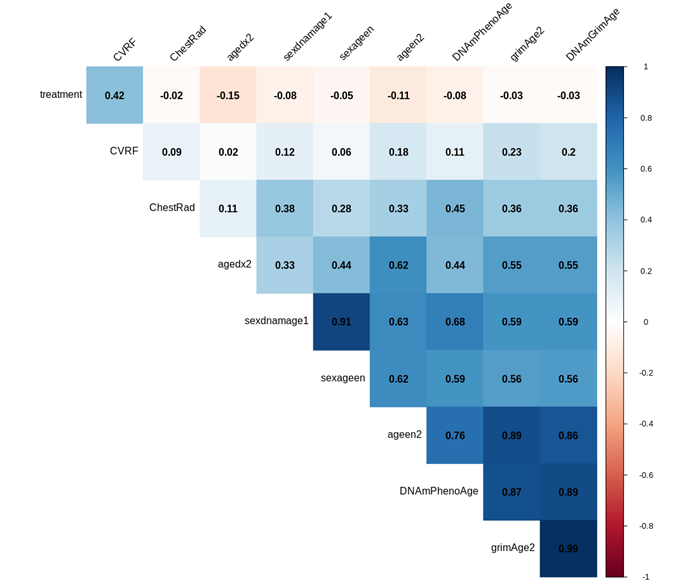** | 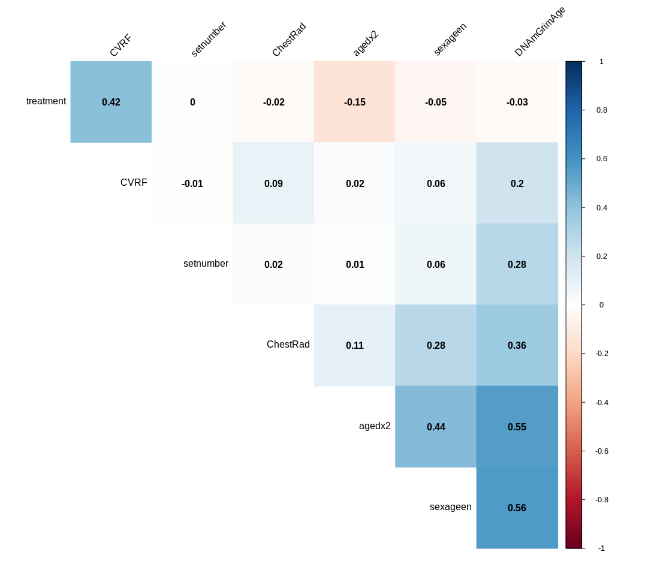 |

**Supplementary Figure 4**. **(A) Heatmap of Pearson correlation coefficient matrix**. The correlation matrix shows the values of the Pearson correlation coefficient for all variables. Positive values are in dark blue and negative in red. Correlation ranges from − 1 to 1, whereby − 1 means a perfect negative linear relationship between variables, 1 indicates a perfect positive linear relationship between variables and 0 indicates that there is no relationship between studied variables. Each square reports a Pearson correlation coefficient. The studied variables were cardiomyopathy (treatment), CVRF, Chest Radiation Y/N (ChestRad), age at the time of primary cancer diagnosis squared (Agedx2), interaction between sex and methylation age (sexdnamage1), interaction term of sex and age at the time of study enrollment (sexageen), age at the time of study enrollment squared (ageen2), methylation pheno age (DNAmPhenoAge), methylation grim age squared (grimAge2), and methylation grim age (DNAmGrimAge).

**(B)** **Heatmap of Pearson correlation with selected variables**. To avoid multi-collinearity in the regression analysis, covariates with correlation coefficient >0.7, in Figure 4A were excluded. Final covariates included in the EWAS model were Cardiomyopathy (treatment), CVRF, matching pair identifier (setnumber), chest radiation (ChestRad), age at primary cancer diagnosis squared (agedx2), the interaction term between sex and age at enrollment (sexageen) and methylation grim age (DNAmGrimAge). CVRF was included due to its known association with cardiomyopathy. The quadratic age term and the interaction between age and sex were considered because of the known non-linear relationship between age and CpG methylation (1-5), as well as the relationship between sex and CpG methylation (6-8), and because cardiomyopathy (main explanatory variable) is also known to be associated with age and sex. Hence, the inclusion of these control variables is biologically and statistically relevant.

**(A)** *EXOC6B*

**
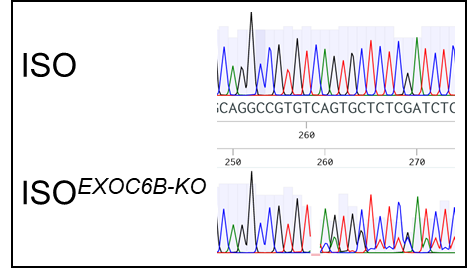
**

**(B)** *FCHSD2*

**
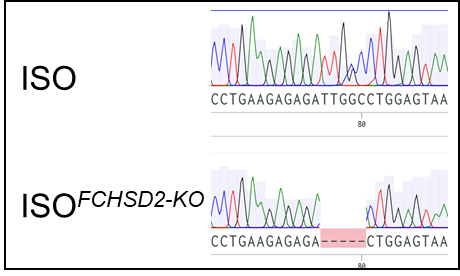
**

**(C)** *NIPAL2*

**
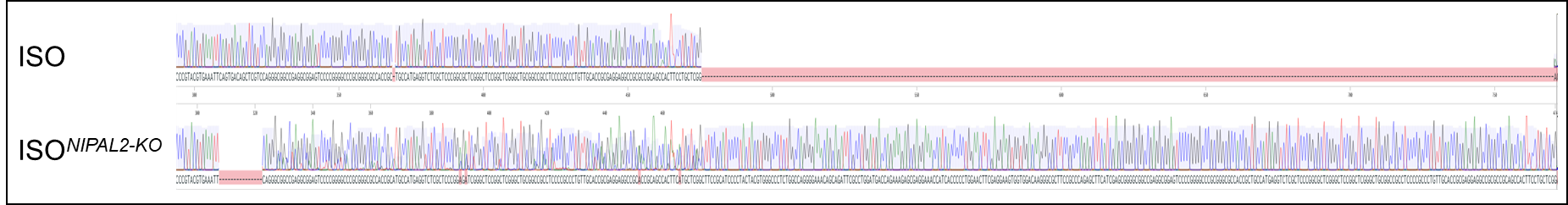
**

**
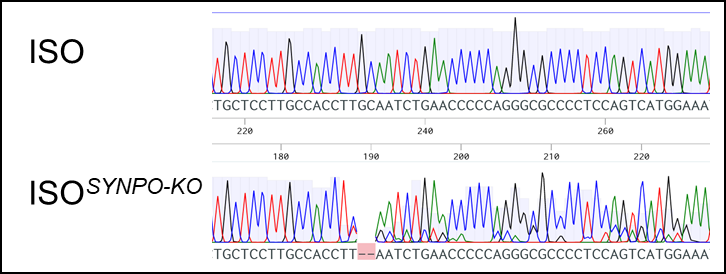
(D)** *SYNPO2*

**(E)** *PDXK*

ISO

ISO*^PDXK-KO^*


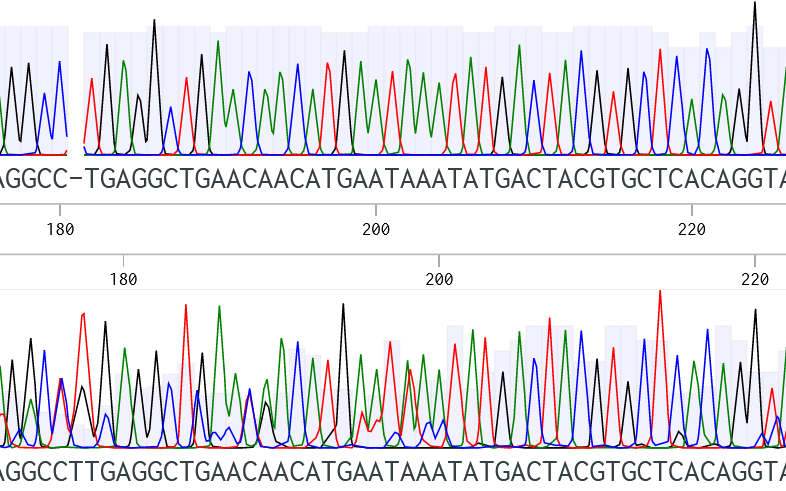


**(F)** *SLMAP*

ISO

ISO*^SLMAP-KO^*


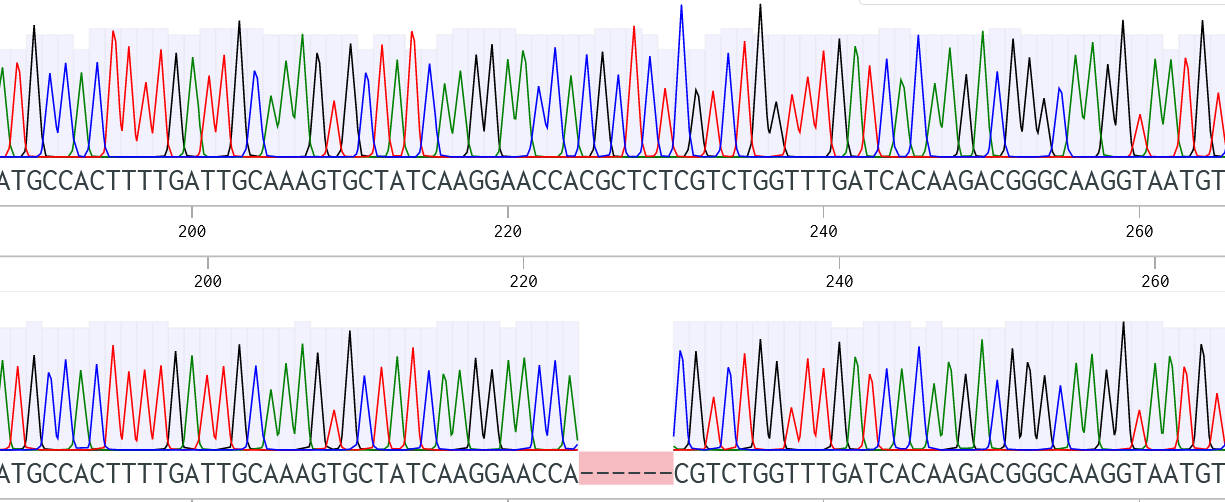


**(G)** *PNPO*


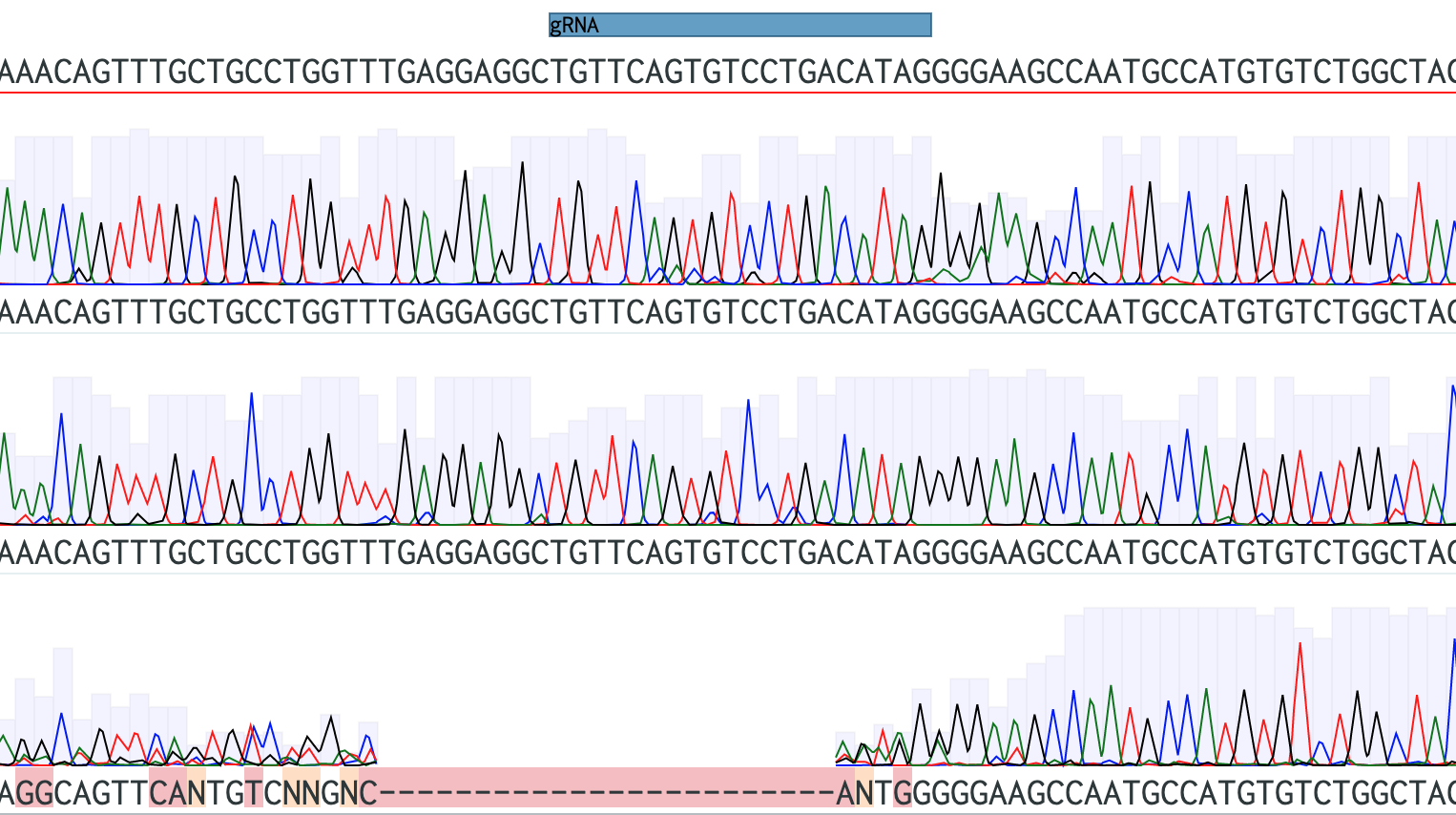


ISO

ISO*^PNPO-KO^*

**(H)** *RORA*


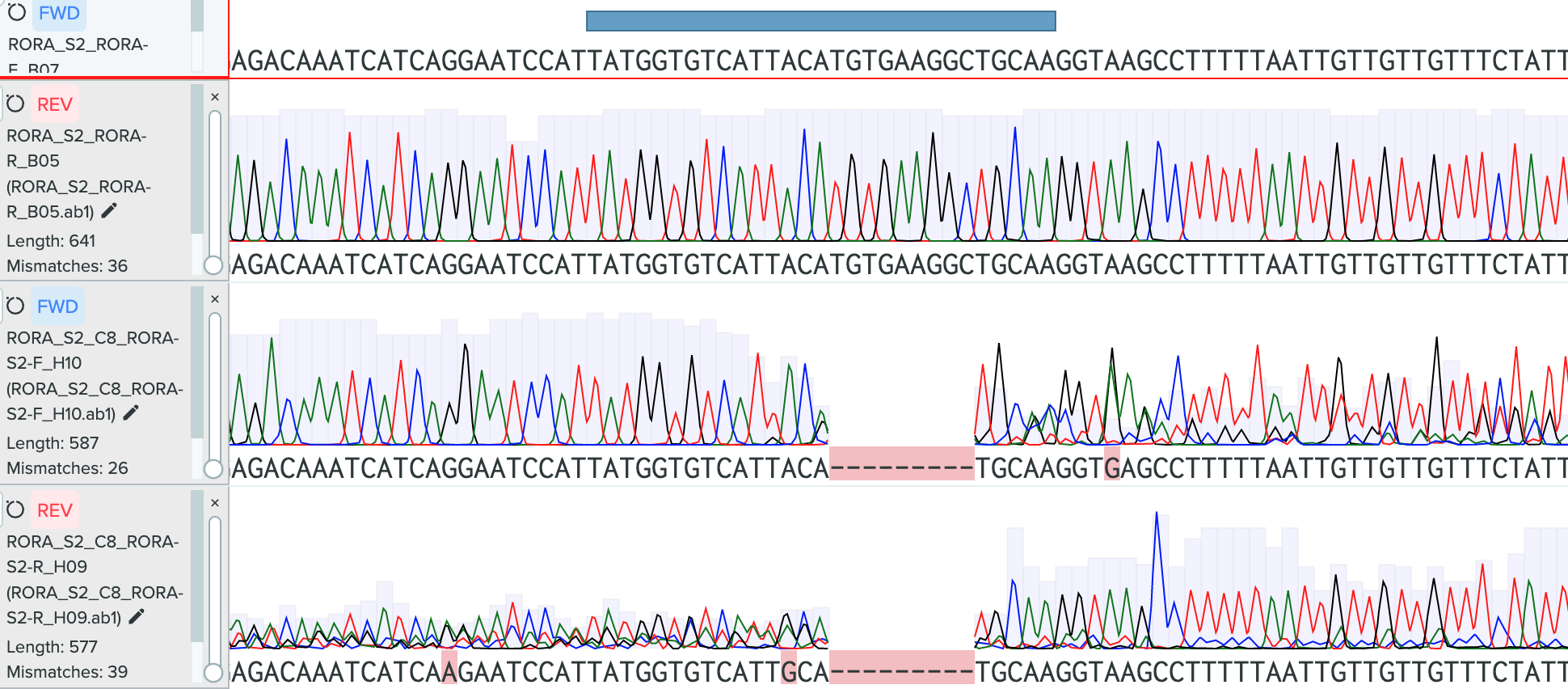


ISO

ISO*^RORA-KO^*

**Supplementary Figure 5.** Validation of CRISPR/Cas9–mediated knockout for genes **(A)** ***EXOC6B*** KO Exon 1, clone 45, 1 base-pair deletion **(B) *FCHSD2*** KO Exon 4, clone 12, 5 base-pair deletion **(C) *NIPAL2*** KO Exon 1, clone 9, 15 base-pair deletion and 295 base-pair insertion **(D) *SYNPO2*** KO Exon 1, clone 40, 2 base-pair deletion **(E) *PDXK*** KO Exon 3, clone 2, 1 base-pair insertion **(F) *SLMAP*** KO Exon 2, clone 41, 6 base-pair deletion **(G) *PNPO*** KO Exon 2, clone 1, 24 base-pair deletion, and **(H) *RORA*** KO Exon 2, clone 8, 9 base-pair deletion in an isogenic human induced pluripotent stem-cell line by Sanger sequencing.


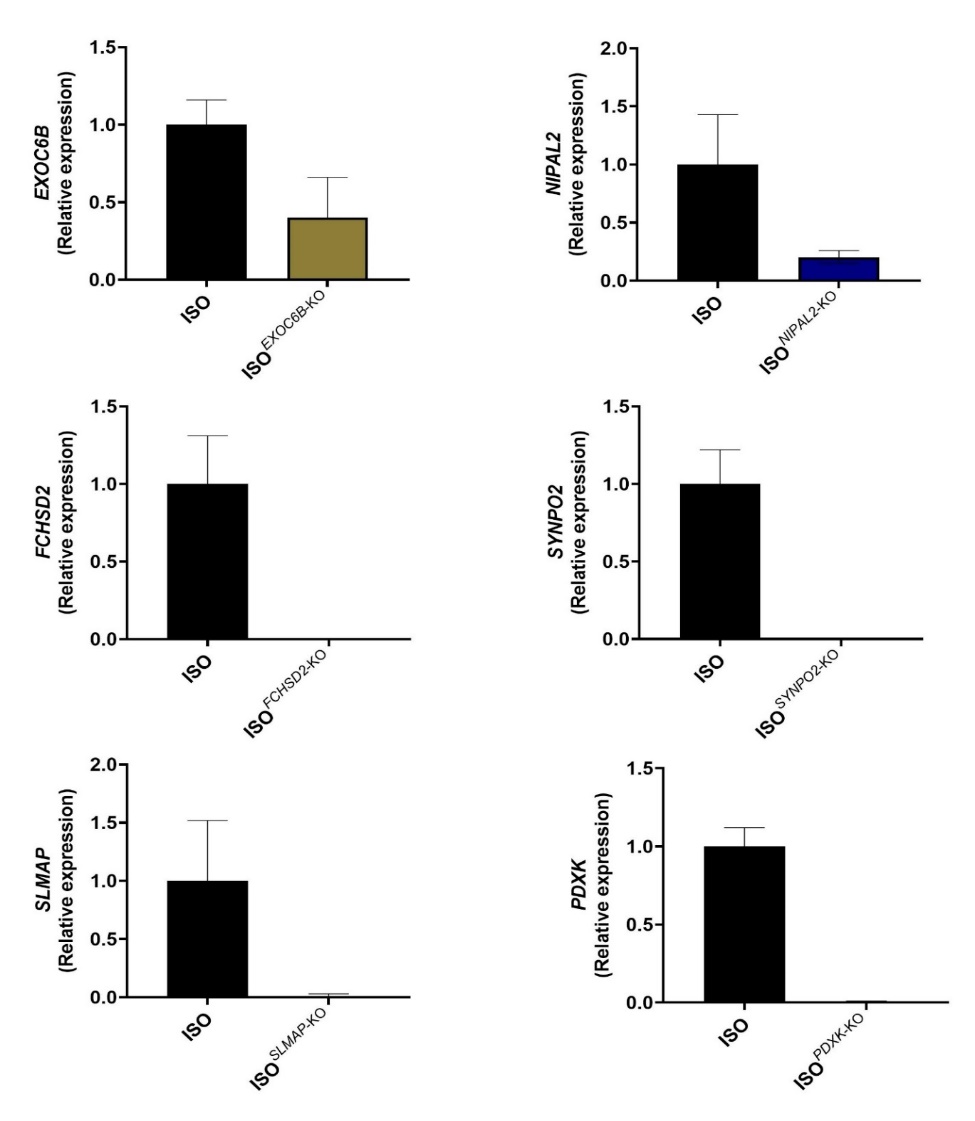

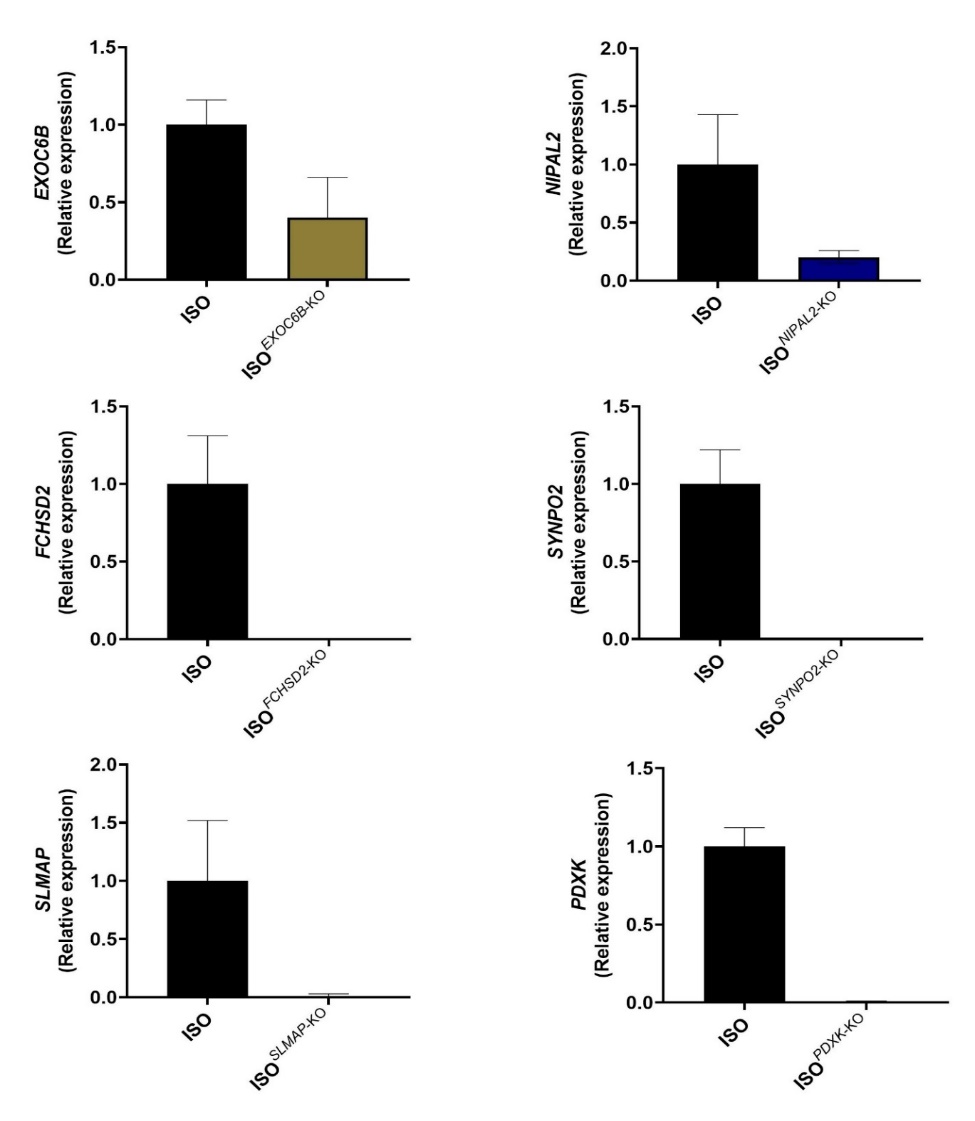

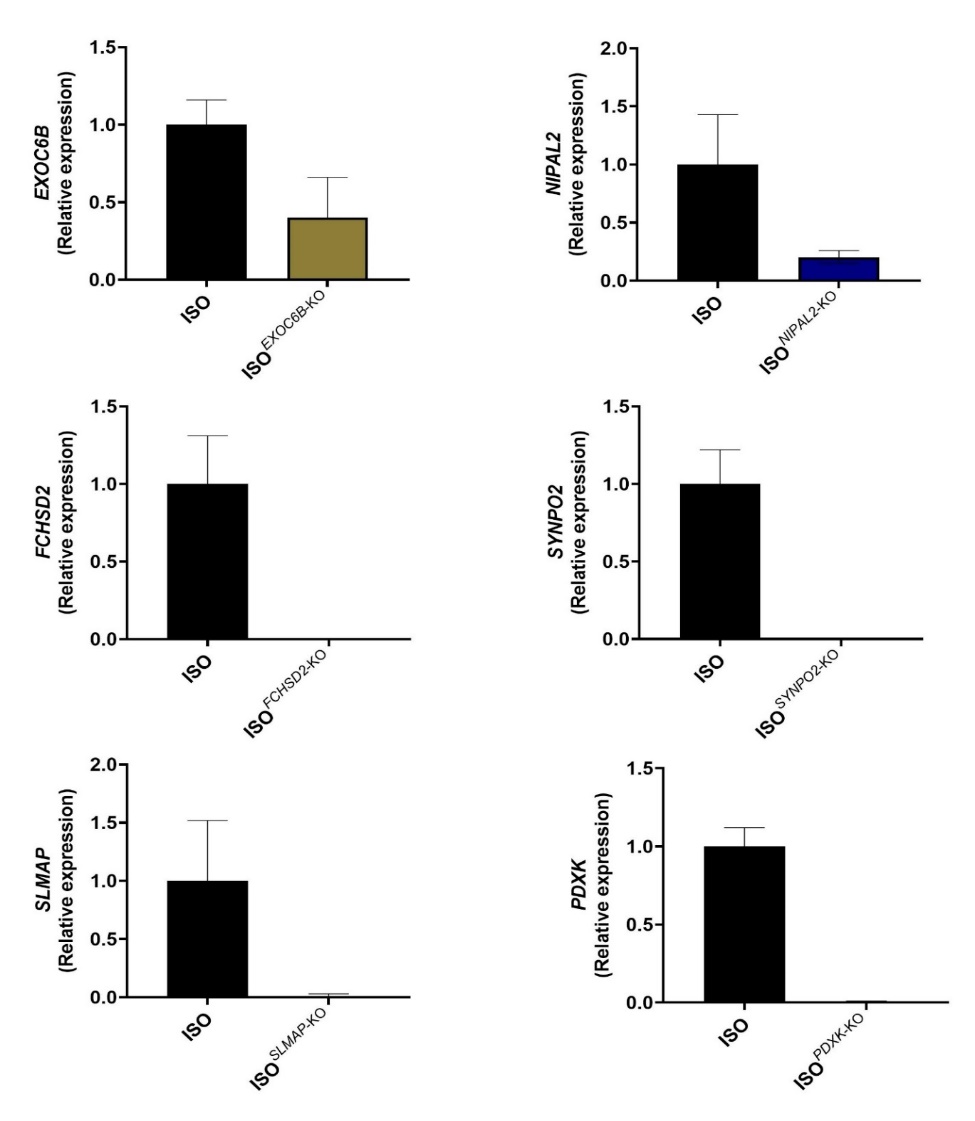

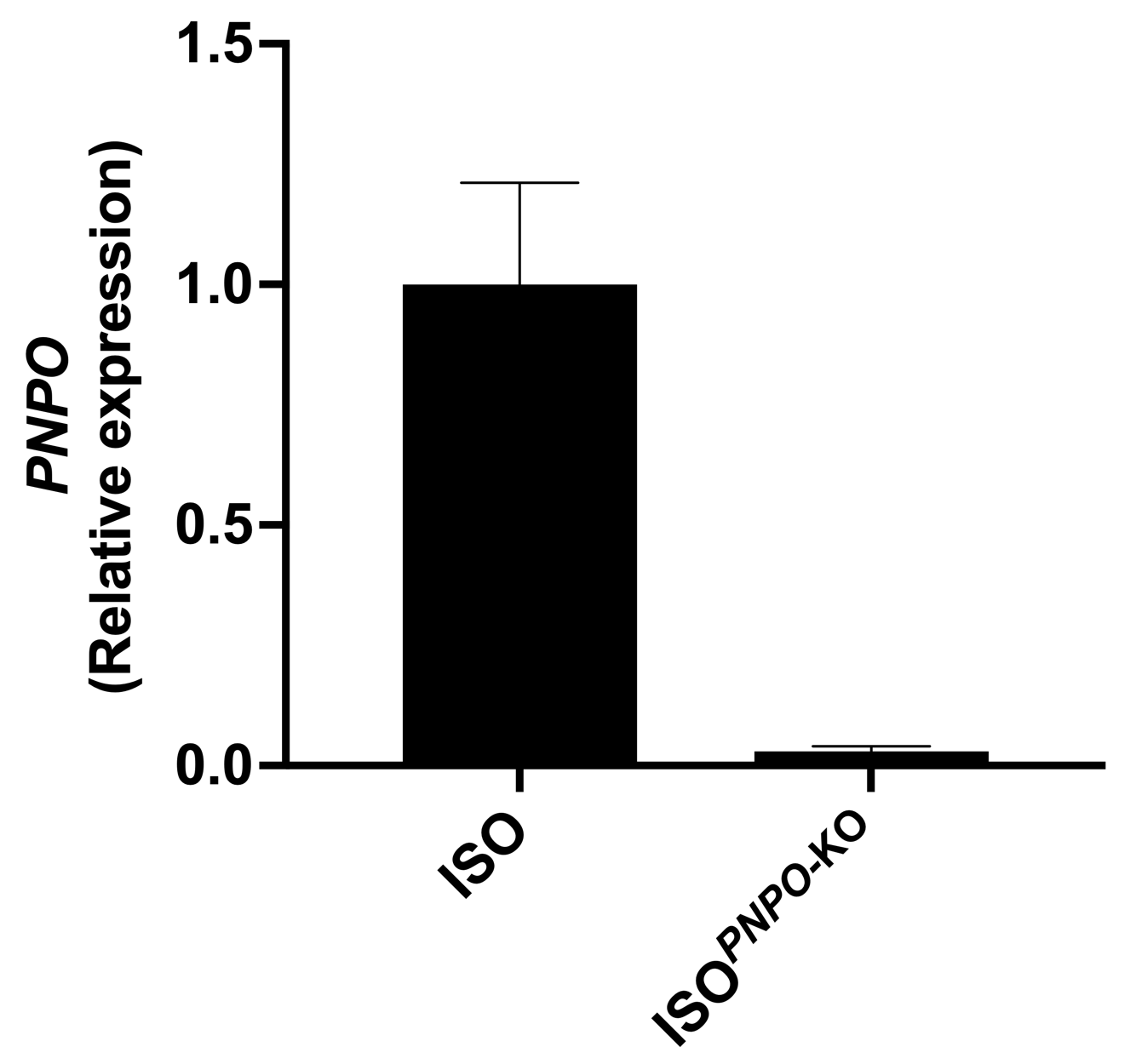

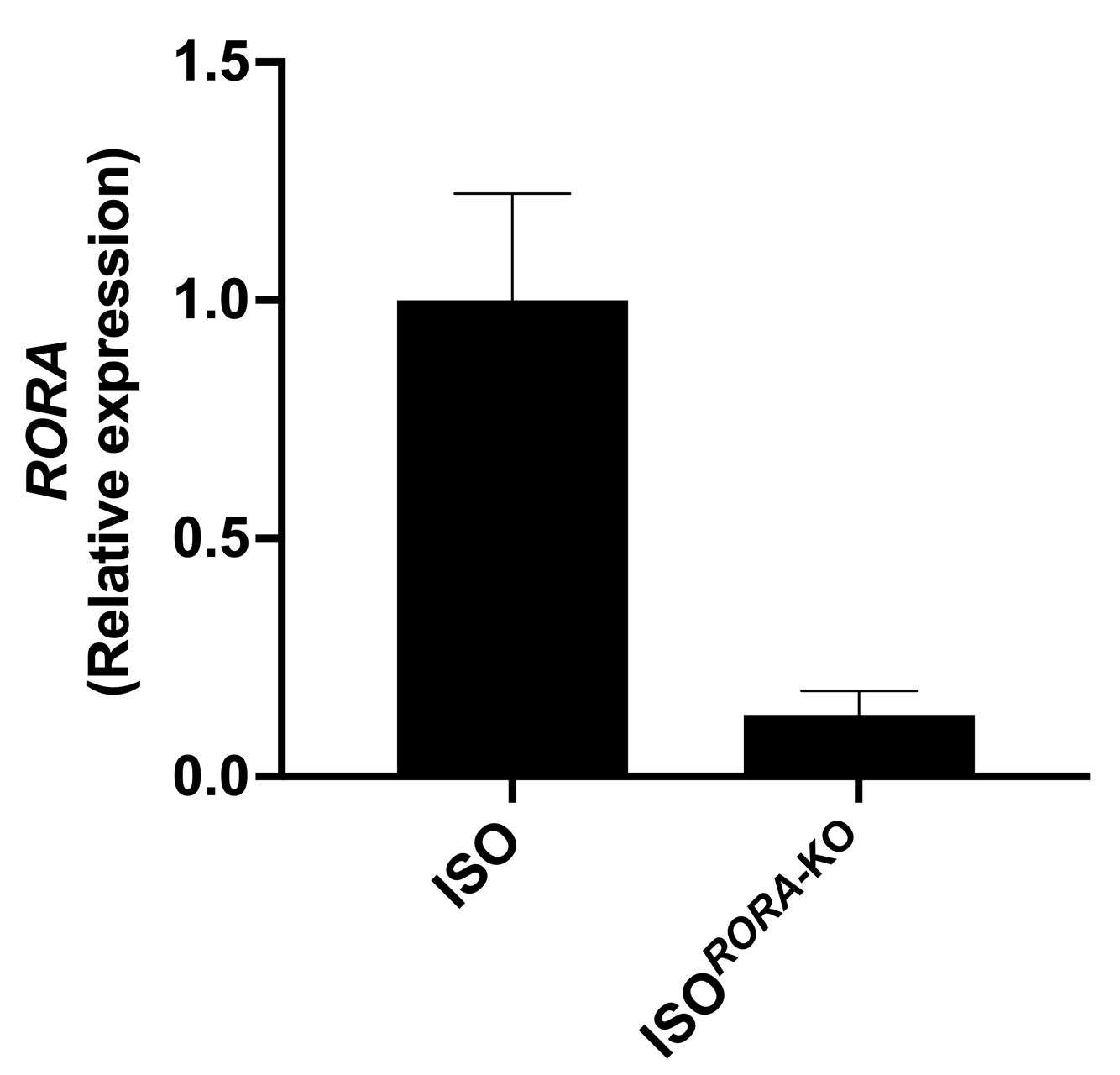


**Supplementary Figure 6**. Validation of target gene knockout by qRT-PCR and analysis of relative mRNA expression for *EXOC6B*, *NIPAL2*, *FCHSD2*, *SYNPO2*, *PDXK*, *SLMAP, PNPO,* and *RORA* in hiPSCs

| City of Hope National Medical Center |
| --- |
| Children's Medical Center Dayton |
| Helen DeVos Children's Hospital |
| Indiana University - Riley Childrens Hospital |
| C.S. Mott Children's Hospital |
| University of Minnesota Cancer Center |
| CancerCare Manitoba |
| Memorial Sloan Kettering Cancer Center |
| Childrens Hospital London Health Sciences |
| Childrens Hospital of Philadelphia |
| University of Pittsburgh |
| Princess Margaret Hospital for Children |
| Seattle Children's |
| UCLA David Geffen School of Medicine |
| British Columbia's Children's Hospital |
| Children's Healthcare of Atlanta, Emory University |
| Children's Hospital of Michigan |
| Stollery Children's Hospital |
| Texas Children's Cancer Center at Baylor College of Medicine |
| Wake Forest University School of Medicine |
| McGill Univ Health Ctr - Montreal Children's Hosp |
| St. Jude Children's Research Hospital Memphis |
| Stanford University Medical Center |
| SUNY Upstate Medical University |
| UT Southwestern Medical Center |
| University of Florida |
| Washington University Medical Center |
| Yale University School of Medicine |
| Rady Children's Hosp San Diego |
| Emanuel Hospital-Health Center |
| Tulane University Medical Center |

**List of Participating Institutions**

**Supplementary Table 1A**: gRNAs with ligation overhangs used for generating CRISPR/Cas9 knockouts

| **Target Gene** | **gRNA forward sequence** | **Reverse complement** |
| --- | --- | --- |
| ***PDXK*** | CACCGAGCTCCAGGAGTTGTACGA | aaacTCGTACAACTCCTGGAGCTC |
| ***FCHSD2*** | CACCGTACCTGAAGAGAGATTGGCC | aaacGGCCAATCTCTCTTCAGGTAC |
| ***NIPAL2*** | CACCGCAGTGACAGCTCGTCCAGGG | aaacCCCTGGACGAGCTGTCACTGC |
| ***SYNPO2*** | CACCGTGGGGGTTCAGATTGCAAGG | aaacCCTTGCAATCTGAACCCCCAC |
| ***EXOC6B*** | CACCGAGAGATCGAGAGCACTGACA | aaacTGTCAGTGCTCTCGATCTCTC |
| ***SLMAP Exon2***  **SLMAP Exon18** | CACCGATCAAACCAGACGAGAGCG | aaacCGCTCTCGTCTGGTTTGATC |
|  | CACCGCAGCAAAGGTTGCCTCTGAG | aaacCTCAGAGGCAACCTTTGCTGC |
| ***PNPO*** | CACCGTGTTCAGTGTCCTGACATAG | aaacCTATGTCAGGACACTGAACA |
| ***RORA*** | CACCGTATGGTGTCATTACATGTGA | aaacTCACATGTAATGACACCATA |

**Supplementary Table 1B**: PCR primers used to validate CRISPR/Cas9-mediated genome editing by Sanger sequencing.

| ***PDXK*** | Exon3- F | 5' CCCCATGGCTTCCTCTGCCTCT 3' |
| --- | --- | --- |
|  | Exon3- R | 5' GCACAGCCTGGGCAGTCATTGT 3' |
| ***FCHSD2*** | Exon4- F | 5' AAGGAAAAGAGCTTCCTTTATGG 3' |
|  | Exon4- R | 5' CCGTTTCTAAAGAAAATAGGCAAA 3' |
| ***NIPAL2*** | Exon1- F | 5' CCGAGCAGGAAGTGGCTGC 3' |
|  | Exon1- R | 5' CGGGTGGCTGAGGTCAAG 3' |
| ***SYNPO2*** | Exon1- F | 5' CGCACAAATTCGCAGCAGG 3' |
|  | Exon1- R | 5' GCCCATTACTCTCCAAAGTTTCTTA 3' |
| ***EXOC6B*** | Exon1- F | 5' TGTCGAGCCTGAGGGAGG 3' |
|  | Exon1- R | 5'  CAGCCTTCGTTCTCCCTCC 3' |
| ***SLMAP*** | Exon2- F | 5’ GCTTGTCTTCCCACCCAAGT 3' |
|  | Exon2- R | 5' CGCAAAGCCACGAATCAACT 3' |
| ***PNPO*** | Exon2- F | 5' ACCTGTAAAATGGGGCACAG 3' |
|  | Exon2- R | 5' GTGGCCTGGTCTTTGGTTTA 3' |
| ***RORA*** | Exon2- F | 5' CTGAGGCCTAAACTGCGAAC 3' |
|  | Exon2- R | 5' TTGCCACAGAGATTGCTCAT 3' |

**Supplementary Table 2**: qRT-PCR TaqMan probes used for verification of CRISPR/Cas9-mediated genome editing

| **Gene** | **Taqman primers and probe (Thermofisher)** |
| --- | --- |
| ***PDXK*** | *Hs00177600* |
| ***FCHSD2*** | *Hs01090386* |
| ***NIPAL2*** | *Hs01122391* |
| ***SYNPO2*** | *Hs00326493* |
| ***EXOC6B*** | *Hs01099380* |
| ***SLMAP*** | *Hs01058332* |
| ***PNPO*** | *Hs01555111* |
| ***RORA*** | *Hs00536545* |

**Supplementary Results:**


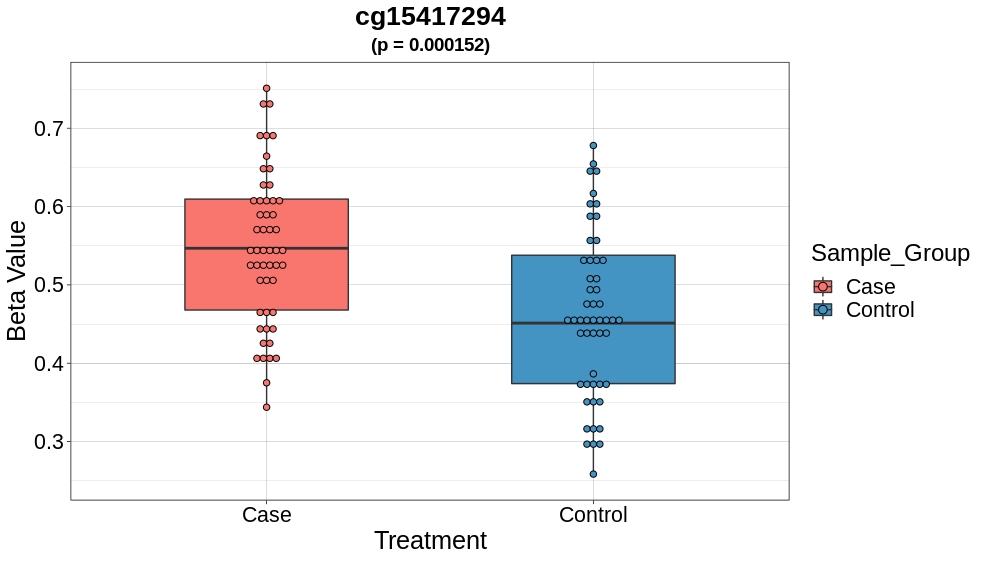
**(A)**


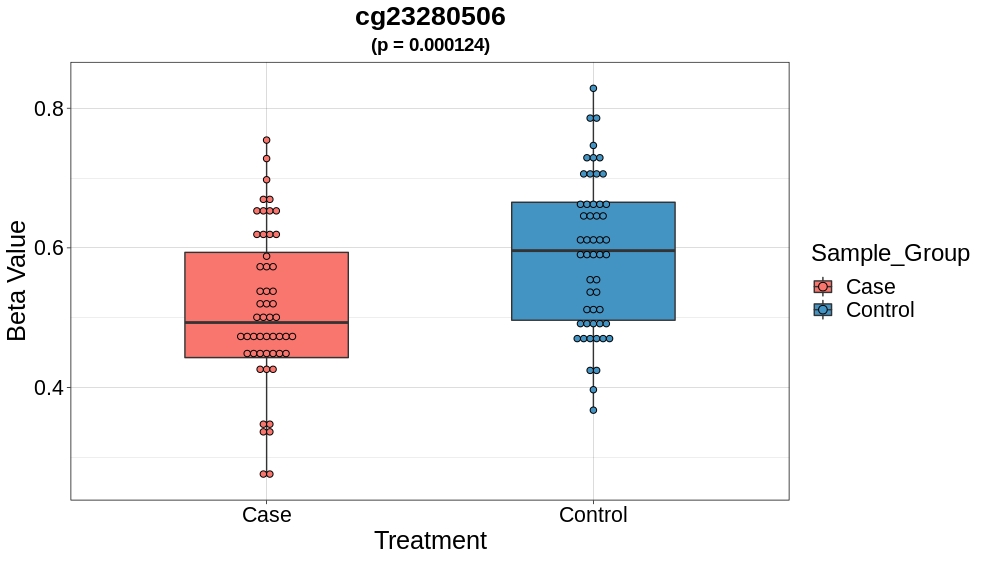
**(B)**

**Supplementary Figure 7.** **Significantly** **differentially methylated probes**. Differences in β values of the highest hyper-methylated and hypo-methylated loci between cases (N=52) and controls (N=52) is shown **(A)** **cg15417294** (located on the body of gene *Slc18A2*) and, **(B)** **cg23280506** (intergenic region on CpG island). Y-axis represents methylation levels (β-values after normalization, 0 = not methylated, 1 = fully methylated). Cases (orange) compared to controls (blue).

**
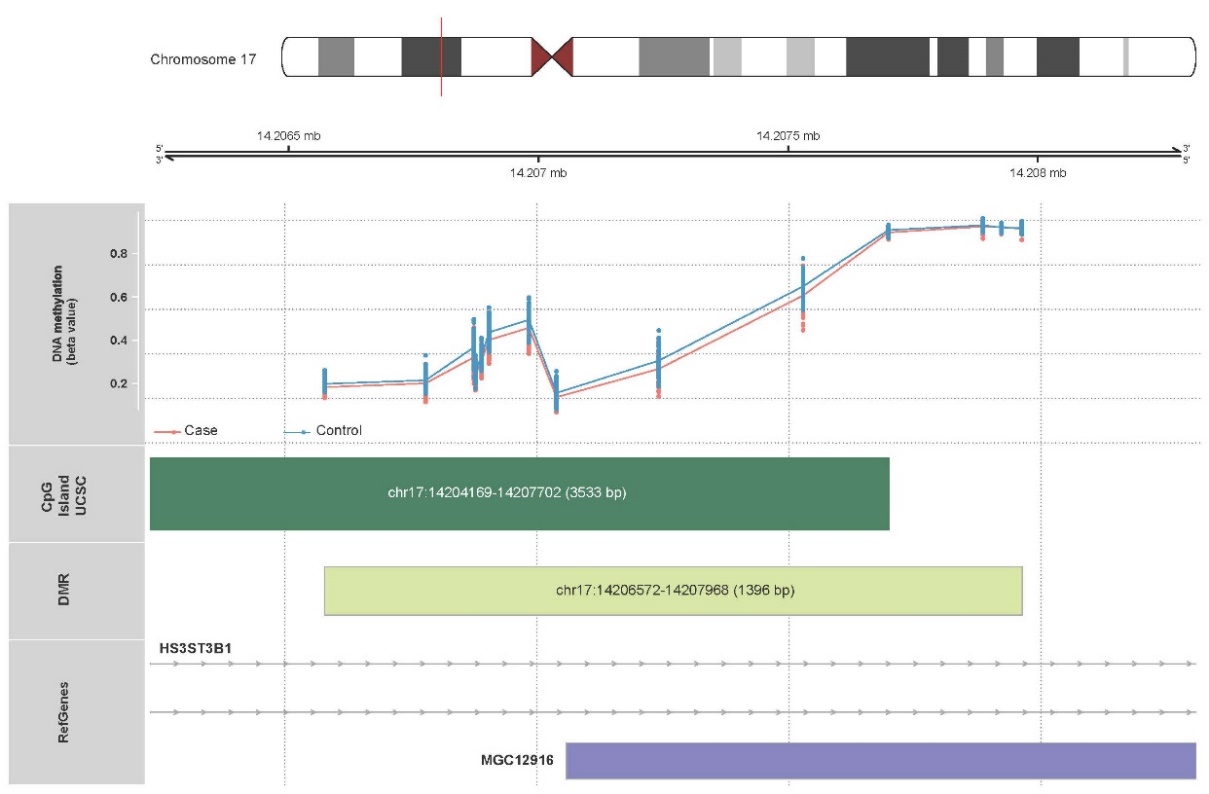

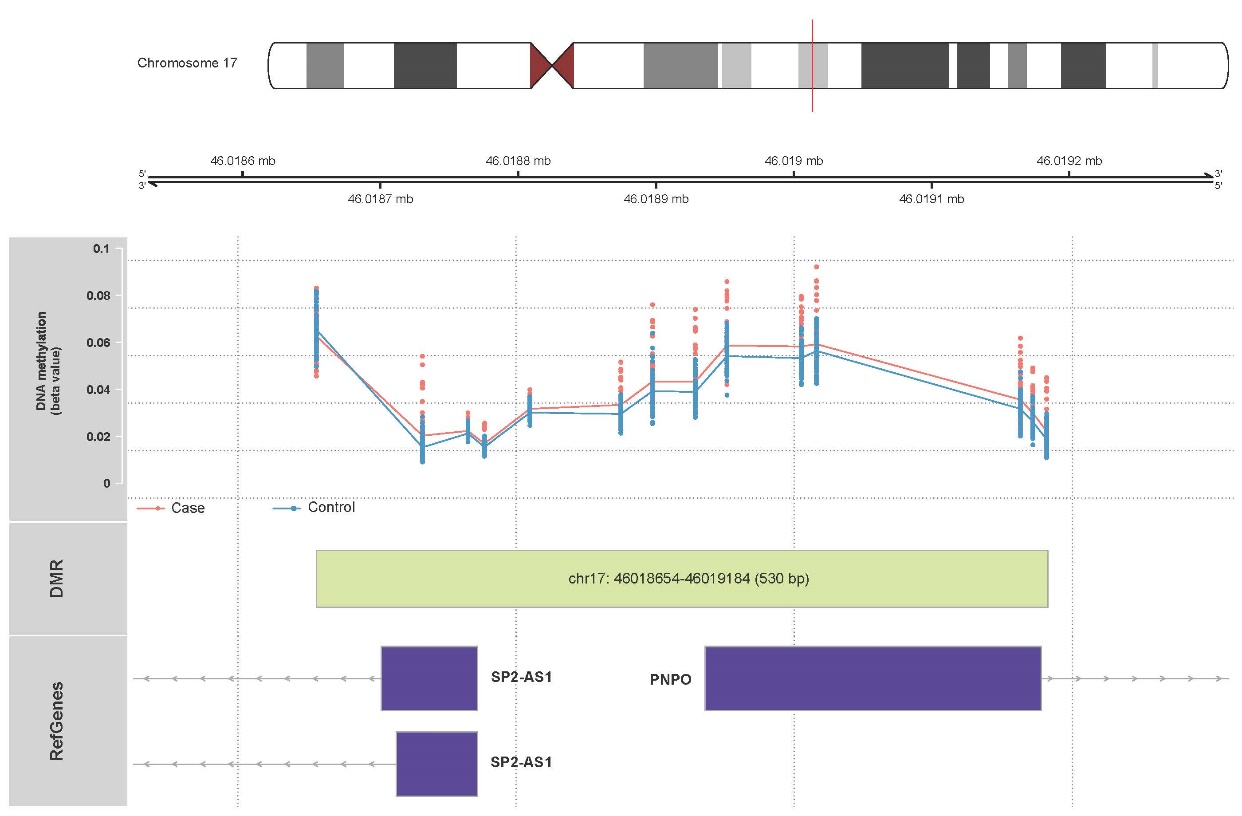
**

**Supplementary Figure 8.** Schematic representation of the top-ranked differentially methylated regions **(A) *HS3ST3B1*** (hypomethylated), and **(B) *PNPO/SP2-AS1*** (hypermethylated). The genomic coordinates are plotted on the top lanes using the version hg19 of the UCSC genome browser. Mean beta values for Cases (orange line) and Controls (blue lines) is plotted on the next lane. The dark green box indicates the CpG island followed by DMRs in light green box. Gene/ exons are shown in dark blue and ncRNA in light blue.

**
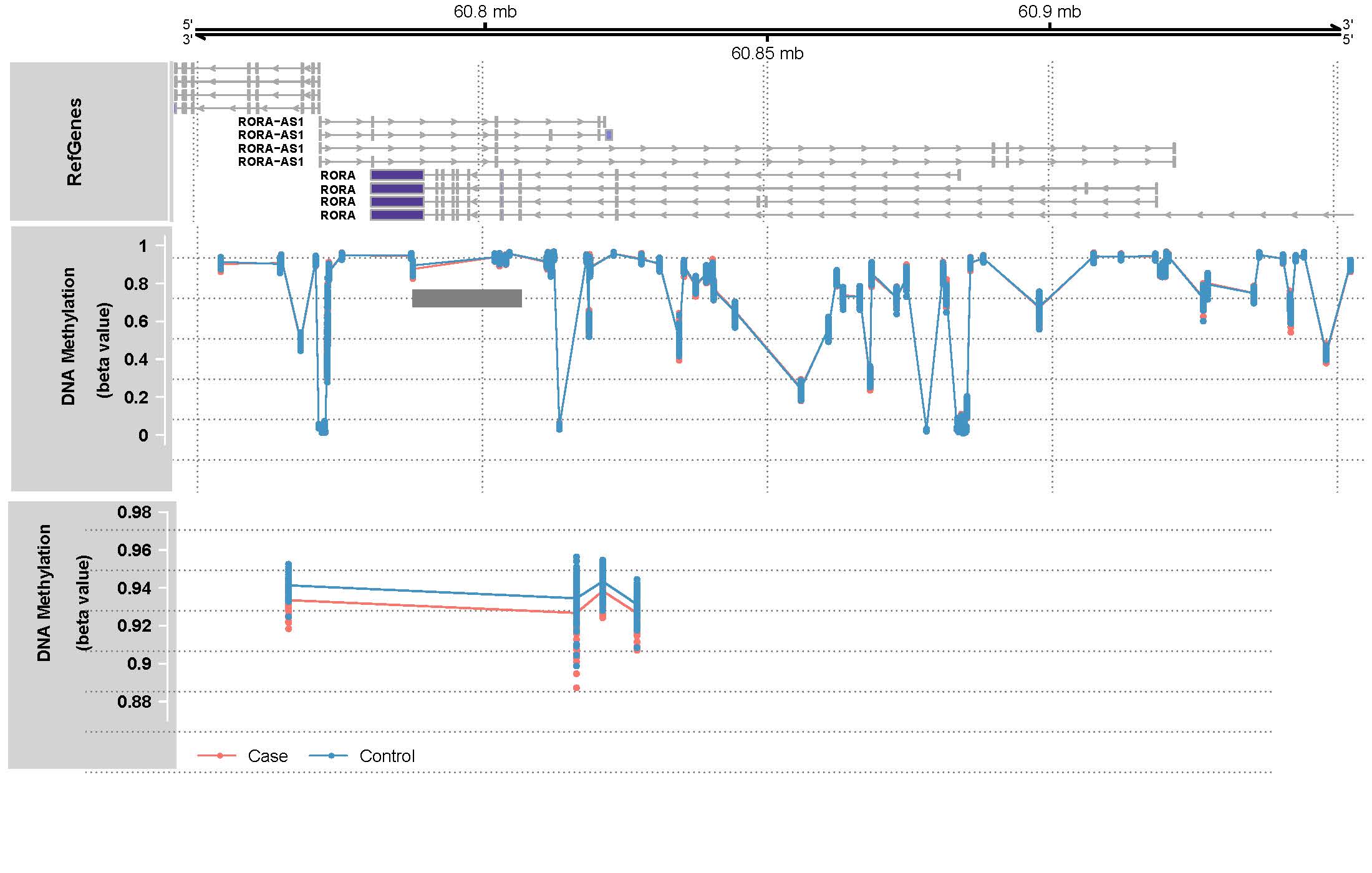
**

**Supplementary Figure 9.** Schematic representation of the top-ranked probe cg15939386 on *RORA-AS1*; *RORA* from the EWAS analysis. The genomic coordinates are plotted on the top lanes using the version hg19 of the UCSC genome browser. Mean beta values for Cases (orange line) and Controls (blue lines) is plotted on the next lane. Gene/ exons are shown in dark blue and ncRNA in light blue. The grey bar shows the zoomed in regions in the lower panel.

**Supplementary References**:

1. Bell JT, Tsai PC, Yang TP, Pidsley R, Nisbet J, Glass D, Mangino M, Zhai G, Zhang F, Valdes A, Shin SY, Dempster EL, Murray RM, Grundberg E, Hedman AK, Nica A, Small KS, Mu TC, Dermitzakis ET, McCarthy MI, Mill J, Spector TD, Deloukas P. Epigenome-wide scans identify differentially methylated regions for age and age-related phenotypes in a healthy ageing population. PLoS Genet. 2012;8(4):e1002629. Epub 2012/04/26. doi: 10.1371/journal.pgen.1002629. PubMed PMID: 22532803; PMCID: PMC3330116.

2. Bollati V, Schwartz J, Wright R, Litonjua A, Tarantini L, Suh H, Sparrow D, Vokonas P, Baccarelli A. Decline in genomic DNA methylation through aging in a cohort of elderly subjects. Mech Ageing Dev. 2009;130(4):234-9. Epub 2009/01/20. doi: 10.1016/j.mad.2008.12.003. PubMed PMID: 19150625; PMCID: PMC2956267.

3. Florath I, Butterbach K, Muller H, Bewerunge-Hudler M, Brenner H. Cross-sectional and longitudinal changes in DNA methylation with age: an epigenome-wide analysis revealing over 60 novel age-associated CpG sites. Hum Mol Genet. 2014;23(5):1186-201. Epub 2013/10/29. doi: 10.1093/hmg/ddt531. PubMed PMID: 24163245; PMCID: PMC3919014.

4. Horvath S, Zhang Y, Langfelder P, Kahn RS, Boks MP, van Eijk K, van den Berg LH, Ophoff RA. Aging effects on DNA methylation modules in human brain and blood tissue. Genome Biol. 2012;13(10):R97. Epub 2012/10/05. doi: 10.1186/gb-2012-13-10-r97. PubMed PMID: 23034122; PMCID: PMC4053733.

5. Langevin SM, Houseman EA, Christensen BC, Wiencke JK, Nelson HH, Karagas MR, Marsit CJ, Kelsey KT. The influence of aging, environmental exposures and local sequence features on the variation of DNA methylation in blood. Epigenetics. 2011;6(7):908-19. Epub 2011/05/28. doi: 10.4161/epi.6.7.16431. PubMed PMID: 21617368; PMCID: PMC3154431.

6. Boks MP, Derks EM, Weisenberger DJ, Strengman E, Janson E, Sommer IE, Kahn RS, Ophoff RA. The relationship of DNA methylation with age, gender and genotype in twins and healthy controls. PLoS One. 2009;4(8):e6767. Epub 2009/09/24. doi: 10.1371/journal.pone.0006767. PubMed PMID: 19774229; PMCID: PMC2747671.

7. Liu J, Morgan M, Hutchison K, Calhoun VD. A study of the influence of sex on genome wide methylation. PLoS One. 2010;5(4):e10028. Epub 2010/04/14. doi: 10.1371/journal.pone.0010028. PubMed PMID: 20386599; PMCID: PMC2850313.

8. Zhang FF, Cardarelli R, Carroll J, Fulda KG, Kaur M, Gonzalez K, Vishwanatha JK, Santella RM, Morabia A. Significant differences in global genomic DNA methylation by gender and race/ethnicity in peripheral blood. Epigenetics. 2011;6(5):623-9. Epub 2011/07/12. doi: 10.4161/epi.6.5.15335. PubMed PMID: 21739720; PMCID: PMC3230547.
